# A terpene nucleoside from *M*. *tuberculosis* induces lysosomal lipid storage in foamy macrophages

**DOI:** 10.1172/JCI161944

**Published:** 2023-03-15

**Authors:** Melissa Bedard, Sanne van der Niet, Elliott M. Bernard, Gregory Babunovic, Tan-Yun Cheng, Beren Aylan, Anita E. Grootemaat, Sahadevan Raman, Laure Botella, Eri Ishikawa, Mary P. O’Sullivan, Seónadh O’Leary, Jacob A. Mayfield, Jeffrey Buter, Adriaan J. Minnaard, Sarah M. Fortune, Leon O. Murphy, Daniel S. Ory, Joseph Keane, Sho Yamasaki, Maximiliano G. Gutierrez, Nicole van der Wel, D. Branch Moody

**Affiliations:** 1 Division of Rheumatology, Immunity and Inflammation, Brigham and Women’s Hospital, Harvard Medical School, Boston, Massachusetts, USA.; 2 Electron Microscopy Centre Amsterdam, Amsterdam University Medical Centre, Amsterdam, Netherlands.; 3 Host-Pathogen Interactions in Tuberculosis Laboratory, The Francis Crick Institute, London, United Kingdom.; 4 Department of Immunology and Infectious Diseases, Harvard T.H. Chan School of Public Health, Boston, Massachusetts, USA.; 5 Department of Molecular Immunology, Research Institute for Microbial Diseases, Osaka University, Suita, Japan.; 6 Department of Clinical Medicine, Trinity Translational Medicine Institute, St. James’s Hospital, Trinity College, Dublin, Ireland.; 7 Department of Chemical Biology, Stratingh Institute for Chemistry, Groningen, Netherlands.; 8 Casma Therapeutics, Cambridge, Massachusetts, USA.

**Keywords:** Infectious disease, Microbiology, Macrophages, Tuberculosis

## Abstract

Induction of lipid-laden foamy macrophages is a cellular hallmark of tuberculosis (TB) disease, which involves the transformation of infected phagolysosomes from a site of killing into a nutrient-rich replicative niche. Here, we show that a terpenyl nucleoside shed from *Mycobacterium tuberculosis*, 1-tuberculosinyladenosine (1-TbAd), caused lysosomal maturation arrest and autophagy blockade, leading to lipid storage in M1 macrophages. Pure 1-TbAd, or infection with terpenyl nucleoside–producing *M. tuberculosis*, caused intralysosomal and peribacillary lipid storage patterns that matched both the molecules and subcellular locations known in foamy macrophages. Lipidomics showed that 1-TbAd induced storage of triacylglycerides and cholesterylesters and that 1-TbAd increased *M. tuberculosis* growth under conditions of restricted lipid access in macrophages. Furthermore, lipidomics identified 1-TbAd–induced lipid substrates that define Gaucher’s disease, Wolman’s disease, and other inborn lysosomal storage diseases. These data identify genetic and molecular causes of *M. tuberculosis*–induced lysosomal failure, leading to successful testing of an agonist of TRPML1 calcium channels that reverses lipid storage in cells. These data establish the host-directed cellular functions of an orphan effector molecule that promotes survival in macrophages, providing both an upstream cause and detailed picture of lysosome failure in foamy macrophages.

## Graphical abstract

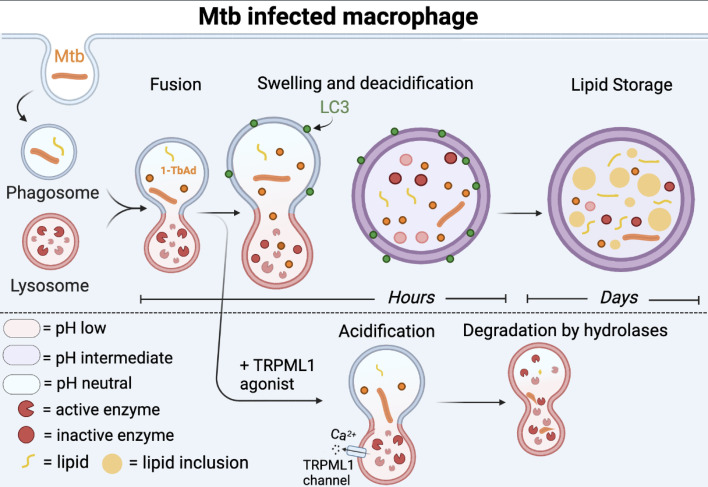

## Introduction

To benefit from immune cloaking and gain access to nutrients within host cells, intracellular bacteria must evade, withstand, or defeat host killing. Bacteria enter macrophages through phagocytosis, where infected phagosomes mature through fusion with lysosomes. Phagosome-lysosome fusion delivers vacuolar ATPases (v-ATPases) that translocate protons to acidify infected compartments (1). Acid pH is the central regulator of phagosomal maturation, which activates approximately 60 downstream pH-dependent enzymes (2) that control antibacterial killing and catabolism of nutrients that would be otherwise available to the pathogen (3). Maturing phagolysosomes also fuse with autophagosomes that express light-chain 3 (LC3) (4, 5) for catalytic digestion of pathogens via autophagy (xenophagy) (6, 7). Thus, phagosomal maturation and autophagy are 2 intersecting pathways that control intracellular bacterial replication.

Among intracellular bacteria, *Mycobacterium tuberculosis* is the most prevalent and deadly pathogen of humans (8). This fact creates a strong rationale to understand *M. tuberculosis*–specific molecules that might confer virulence. *M. tuberculosis* replicates within the phagolysosomal network of macrophages (9), which is a key pathological hallmark of natural tuberculosis (TB) disease (10). Thus, a major advance resulted from early observations that *M. tuberculosis* survives in what is normally a site of pathogen killing via partial blockade of phagosome-lysosome fusion (11). Whereas fully mature lysosomes generate a pH of approximately 5, *M. tuberculosis* resets the lysosomal pH to approximately 6.2 (12). *M. tuberculosis* infection also triggers autophagy (13), which controls mycobacterial clearance in some situations (14), but not others (15). However, the *M. tuberculosis*–specific mycobacterial genes and molecules that cause lysosomal failure, as well as therapeutic agents that could reverse this cellular process, remain elusive.

A major cellular hallmark of TB disease is the generation of “foamy” macrophages. “Foamy” originally referred to the hazy appearance of lipid-laden macrophages in atherosclerotic disease (16). Experimentally, foamy cells could be induced by chloroquine, a drug that inhibits acidification to inactivate lysosomal hydrolases, including lysosomal acid lipase (LAL), which is required for cholesterol ester catalysis (17). Decades later, the term “foamy” was widely applied to *M. tuberculosis*–infected macrophages because they phenocopy the visible lipid overload through accumulation of neutral lipids in phagosomes (18), perilipin-coated lipid droplets (19), or both (20). Foamy macrophage induction by *M. tuberculosis* prominently involves triacylglycerols (TAGs) and cholesterylesters (CEs) (21, 22), which is like “setting up a fast-food joint for unexpected guests” (23). Bacteria-induced lipid storage is likely physiologically significant because TAG and CE access promotes *M. tuberculosis* survival in macrophages (24) and persistence in vivo (25).

Complementing genetic approaches, comparative metabolomic screens can identify candidate virulence factors that are present in *M. tuberculosis* and absent in mycobacterial species that fail to infect or transmit. A subtractive screen of *M. tuberculosis* versus bacillus Calmette-Guérin (BCG) discovered a terpene nucleoside, 1-tuberculosinyladenosine (1-TbAd) (26), which is normally produced at high levels (27, 28). The 1-TbAd biosynthesis genes (*Rv3377c-Rv3378c*) appeared early in the evolution of the *M. tuberculosis* complex (MTBC), so 1-TbAd expression correlates with evolved virulence (28–30). Yet, 1-linked purines are rare in nature, so 1-TbAd is an orphan molecule that is chemically unrelated to bacterial immunogens, toxins, or quorum sensors that might inform its function. Starting from first chemical principles, 1-TbAd is composed of a halimane lipid core linked to adenosine, creating an amphipathic conjugate base, which are the 2 chemical properties of lysosomotropic drugs (31). 1-TbAd crosses membranes into acidic liposomes to raise pH (32), suggesting the cell biological hypothesis that is tested here: 1-TbAd is a naturally shed antacid that locally inhibits host macrophage function during *M. tuberculosis* infection.

Our data show how synthetic and natural terpene metabolites induce a 2-step mechanism of rapid lysosome failure followed by durable patterns of lipid storage in macrophages that recapitulate key aspects of *M. tuberculosis*–infected foamy macrophages and inborn lysosomal storage diseases. Causal linkages are reinforced by data showing that lysosomal swelling and lipid storage are reversed by deletion of terpenyl nucleosides in *M. tuberculosis*, chemical reengineering of the lysosomotropic determinant on 1-TbAd, or by activation of lysosomal function via TRPML1 calcium channels (33). These data define the host-directed functions of an orphan mycobacterial effector molecule and establish a species-specific molecular cause of lysosome failure, autophagy blockade, and growth restriction in *M. tuberculosis*–infected macrophages.

## Results

### 1-TbAd does not detectably activate an immune response.

We asked whether 1-TbAd contributes to the innate immune response of mouse macrophages to *M. tuberculosis* (34). *M. tuberculosis* activates Mincle, TLRs (TLR2), and intracellular NOD2 receptors, which signal via CARD9, MyD88, and NF-κB, respectively (35, 36). To avoid false-positive responses from contaminating microbial lipids, we synthesized authentic 1-TbAd (37). We used WT, *Myd88*^^–^^macrophages, *Card9*^^–^^ macrophages, and NOD2 reporters that respond to cord factor (38), LPS, or muramyl dipeptide (MDP) controls, respectively. 1-TbAd caused no significant response (Supplemental Figure 1, A and B; supplemental material available online with this article; https://doi.org/10.1172/JCI161944DS1), providing evidence against activation of 3 major macrophage signaling pathways. Combined with prior work that failed to find toxin, quorum sensing, or immune activity (26, 28, 32), all screens failed to detect receptor-mediated cellular responses to 1-TbAd.

### Lysosomal remodeling by 1-TbAd.

**Figure 1:**
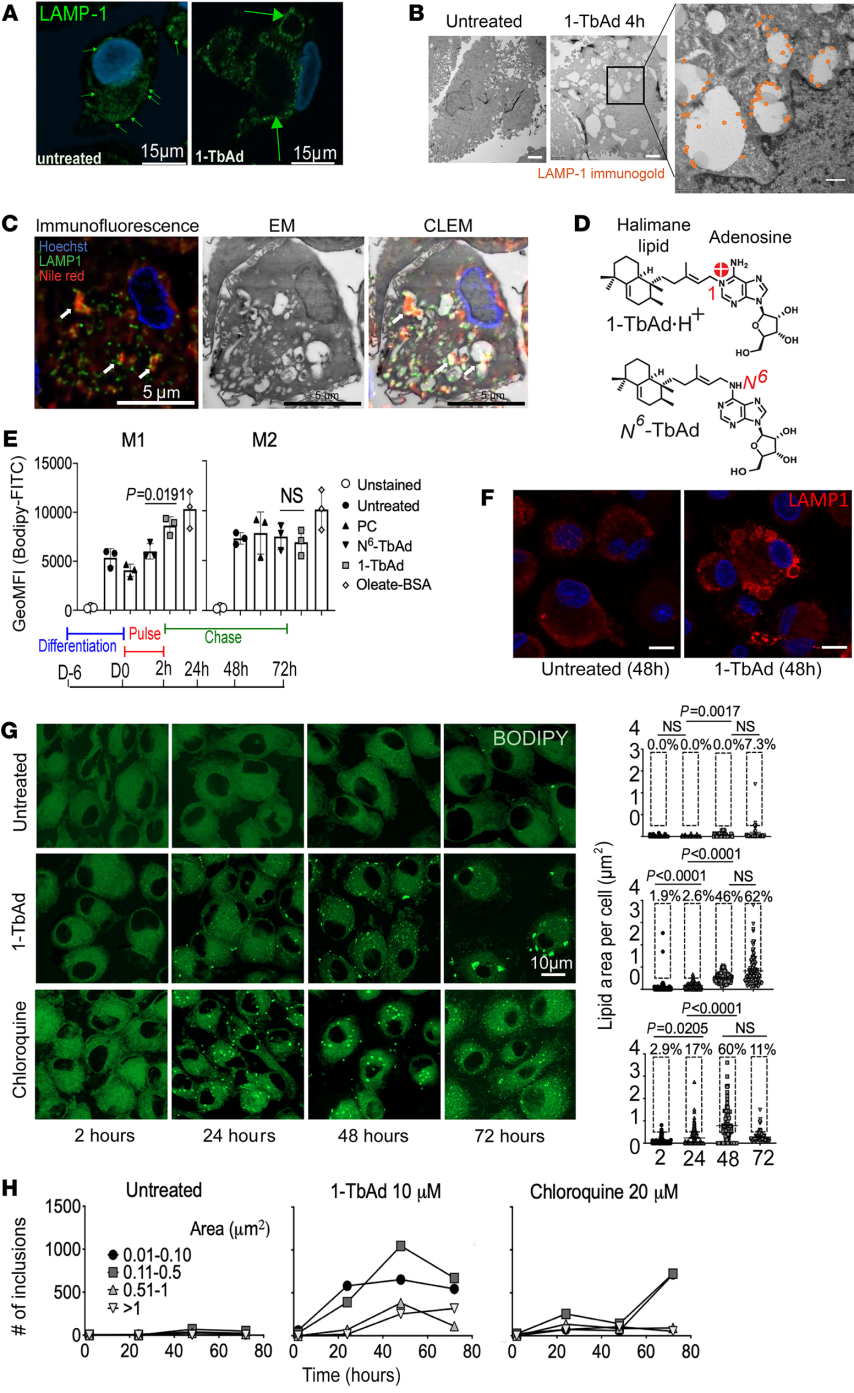
1-TbAd induces swelling of LAMP1 compartments and lipid overload in macrophages. (**A**) LAMP1^+^ lysosomes in human M1 macrophages lacked visible lumina and thus appeared as puncta (small green arrows), but 1-TbAd treatment generated swollen lysosomes that appeared as rings (large green arrows). Scale bars: 15 μm. (**B**) Transmission EM (TEM) of human macrophages stained with LAMP1 immunogold (orange) shows swollen electron-lucent lysosomes with intralysosomal inclusions after treatment with 1-TbAd (10 μM) for 4 hours. Scale bars: 1 μm (left), 2 μm (middle), 500 nm (enlarged inset).(**C**) Macrophages treated as in **B** underwent deconvoluted CLEM. Arrows indicate colocalization of LAMP1 and lipid bodies. Scale bars: 5 μm. (**D**) Synthetic *N*^6^-TbAd is a 1-TbAd isomer that lacks the 1-linkage needed for lysosomotropic action. (**E**) Whole-cell BODIPY staining of monocyte-derived M1 and M2 macrophages treated with the indicated lipid or high-dose oleate-BSA as a positive control for lipid overload. (**F**) Human alveolar macrophages were treated with 10 mM 1-TbAd for 48 hours, leading to conversion of LAMP1 puncta to ringed structures. Scale bars: 5 μm. (**G**) A pulse-chase analysis of BODIPY staining in human M1 macrophages was tracked for total lipids, measured as the area per cell. BODIPY^+^ lipid inclusions were binned by size and tracked separately over time. Scale bar: 10 μm.

We next tested a chemical mechanism known as lysosomotropism, whereby 1-TbAd might directly enter lysosomes, where it is protonated and concentrates to cause lysosomal swelling (32). After treatment with 1-TbAd for 4 hours, lysosomal-associated membrane protein 1–positive (LAMP1^^+^^) puncta were replaced with large (300–2000 nm) LAMP1^^+^^ rings (Figure 1A, large arrows). This lysosomal swelling was dose dependent and seen across cells from 3 donors (Supplemental Figure 2). Similarly, in electron microscopy (EM) analysis, 1-TbAd converted compact, electron-dense lysosomes into large electron-lucent compartments that, despite their atypical morphology, could be assigned as lysosome-derived based on LAMP1 immunogold labeling. Markedly swollen lysosomes were broadly present throughout all cells, and high-magnification images showed intralysosomal particulate inclusions (Figure 1B and Supplemental Figure 3). Correlative light and electron microscopy (CLEM) 48 hours after 1-TbAd exposure showed that intralysosomal particulates overlaid with Nile red, demonstrating their lipidic nature (Figure 1C). Thus, 1-TbAd generated the key outcomes expected of a lysosomotrope (31): it rapidly unraveled the normally compact and multilamellar lysosomes to generate swollen compartments containing lipid.

### The chemical determinant of 1-TbAd action on M1 macrophages.

Lipids accumulate in macrophages during *M. tuberculosis* infections to cause a “foamy” appearance. Whereas foamy macrophages are a hallmark of TB disease (18, 20, 21, 39–41), the mycobacterial genes or molecules that cause this lipid storage effect remain unknown. 1-TbAd emerged as a candidate lipid inducer based on CLEM evidence for intralysosomal lipid storage (Figure 1C), and subsequent BODIPY staining quantitated lipid storage as a disease-relevant outcome. After treatment of M1 and M2 macrophages with an unrelated lipid (phosphatidylcholine [PC]), *N^6^*-TbAd, and 1-TbAd for 72 hours, only 1-TbAd significantly increase BODIPY 493/503 staining in M1 macrophages (Figure 1, D and E), similar to cells treated with oleate-BSA to generate overt lipid overload. The alternate *N*^^6^^-linkage (Figure 1D, red) converts TbAd to a weak base with approximately 200-fold less proton capture, which is predicted to block pH effects and retention in lysosomes. Thus, *N*^^6^^-TbAd is a rigorous control that specifically lacks the lysosomotropic determinant (32, 37). We saw no significant increase in BODIPY staining of human M2-differentiated macrophages (Figure 1E). Selective increases in lipid staining in M1 macrophages rule out the possibility that 1-TbAd itself was the stained lipid.

This unexpected difference led to analysis of human alveolar macrophages (AMs). We observed increased lipid staining after 1-TbAd treatment, but these increases did not reach statistical significance among the limited fresh cells that could be recovered by bronchoalveolar lavage (Supplemental Figure 4A). However, at all doses between 5 and 20 μM of 1-TbAd and in all donors, LAMP1 “rings” broadly replaced puncta within 4 hours in human AMs and persisted over 96 hours (Figure 1F and Supplemental Figure 4, B and C). Thus, lysosomal swelling and lipid storage are related but separable cellular processes that occur over hours and days, respectively. One model can explain all results: swelling corresponds to 1-TbAd entry, pH rise, and lysosomal inactivation, and lipid storage is a secondary consequence that depends on the lipid anabolic-catabolic balance of each cell type. Indeed, recent studies show a higher induction of lipid-metabolizing genes in AMs or M2 macrophages, as compared with lower lipid catabolic activity in interstitial macrophages that are similar to the M1 macrophages studied here (42, 43).

### Pulse-chase studies show durable lipid storage patterns.

Next, we compared 1-TbAd activity with chloroquine, a classical lysosomotropic drug (44). Chloroquine enters lysosomes within 1 hour, gets trapped as a cation, and causes durable intralysosomal swelling (31). If 1-TbAd likewise acts through lysosomal trapping, it might cause durable effects after pulse. A 2-hour pulse of chloroquine (20 μM) or 1-TbAd (10 μM) caused rapid, significant, and durable lipid accumulation over days in M1 macrophages, involving approximately 60% of cells (Figure 1G). We observed that staining patterns were dominated by puncta, rather than the hazy staining patterns typical of membranes, suggesting that lipids accumulated as the cargo of subcellular compartments. Tracking of total lipid signals, total puncta numbers, and puncta size over time provided insight into the mechanism of lipid storage. We found that chloroquine effects were initiated and resolved more quickly, as compared with the slower, stronger, and persistent 1-TbAd effect. For 1-TbAd, small- and medium-sized inclusions (<0.5 μm^^2^^) initially increased in number and then decreased from 48 to 72 hours. From 48 to 72 hours, large and very large puncta, along with the total BODIPY area, increased, suggesting fusion to form large compartments. Thus, a shed lipid from mycobacteria phenocopies the functions of a lysosomotropic drug to generate extensive lipid storage in M1 macrophages, but 1-TbAd is more potent and durable in effect. These observations raised questions about storage compartments, which likely included lysosomes, but might also involve phagosomes, autophagosomes, or lipid droplets.

### 1-TbAd induces autophagosome accumulation.

**Figure 2:**
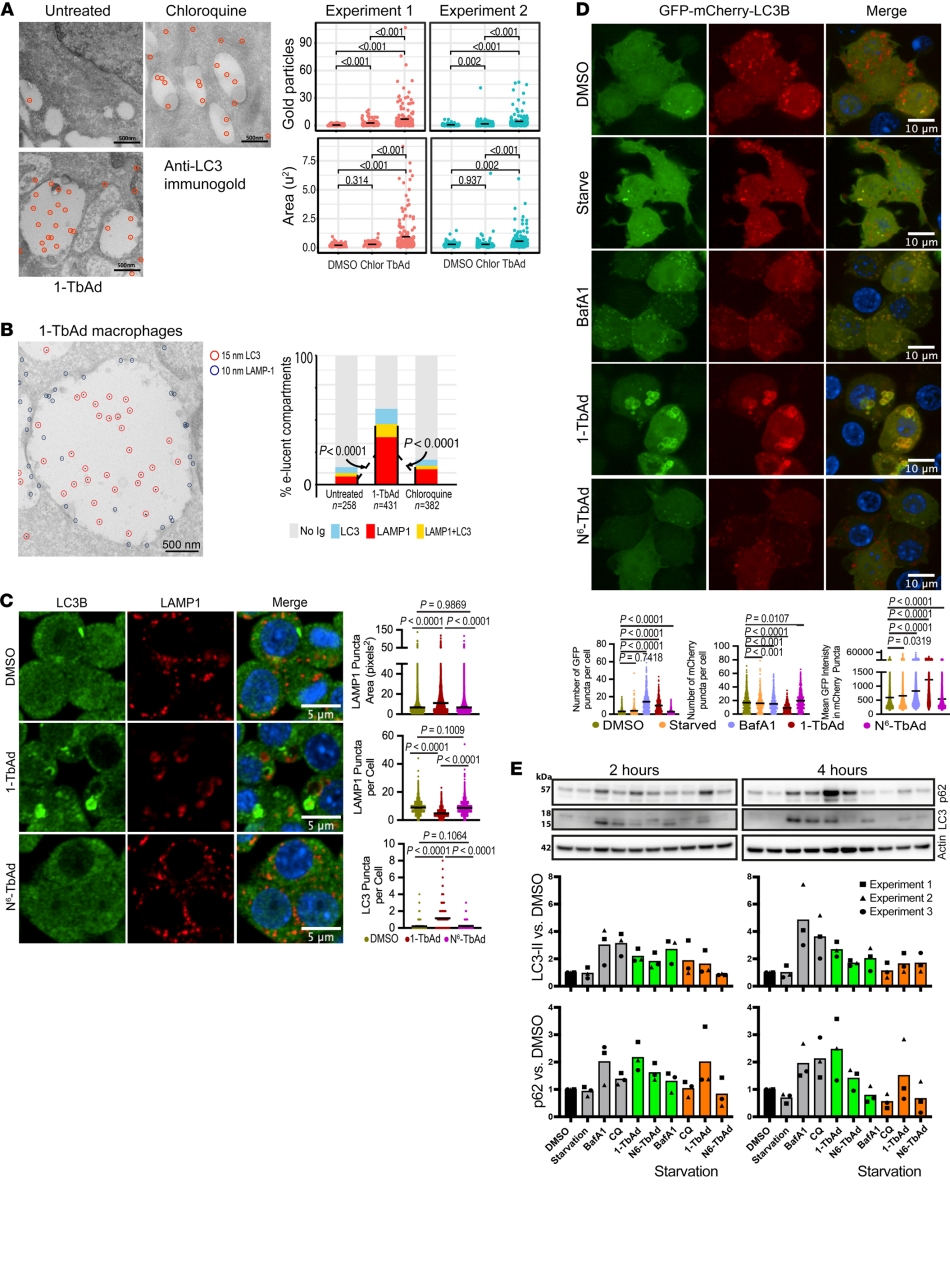
1-TbAd causes the accumulation of autophagosomes due to blockage of autophagic flux. (**A** and **B**) M1 macrophages treated with chloroquine or 1-TbAd (20 μM) for 2 hours were immunogold labeled for LC3, LAMP1, or both markers. The area (μm^2^) of electron-lucent compartments was measured and the number of gold particles were counted per compartment. Double-immunogold labeling was scored as no label (<3 particles) or labeled (>3 particles), with subgroups of LAMP1 single positive, LC3B single positive, and LAMP1 AND LC3B double positive. Single LC3 analysis used a linear model with a negative binomial fit, with *P* values determined by factorial ANOVA and Tukey’s post test. Linear mixed models treated the double label as a random effect variable (χ^2^ *P* << 0.0001). For single and double labels, *P* values were determined by least squares mean post-test after factorial ANOVA and adjustment by Tukey’s method. (**C**) RAW264.7 macrophages stimulated for 4 hours were analyzed by immunofluorescence for LC3B recruitment to LAMP1^+^ compartments. Scale bars: 5 μm. One representative experiment of 3 experiments is shown. *P* values were determined by Browne-Forsythe ANOVA followed by Games-Howell’s multiple comparisons. (**D**) RAW264.7 macrophages transiently expressing GFP-mCherry-LC3B were treated with vehicle (DMSO), BafA1, 1-TbAd, or *N^6^*-TbAd for 4 hours and then fixed. Black bars indicate the mean values and the data are representative of 3 experiments. *P < 0.05, ***P < 0.001, and ****P < 0.0001, by Browne-Forsythe ANOVA followed by Games-Howell’s multiple-comparison test. Scale bars: 10 μm. (**E**) In 3 experiments, RAW264.7 macrophages were stimulated for 2 hours or 4 hours and then subjected to Western blotting.

Given the strong effects of 1-TbAd on lysosomes, we asked if it also inhibited autophagic flux, which requires lysosomes to degrade cargo (45). As seen by EM, 1-TbAd (20 μM) increased the area of the electron-lucent compartments (*P* < 0.001) and showed autophagosome-defining LC3^^+^^ membranes therein (*P* < 0.001) (Figure 2A). These effects were stronger than those seen with chloroquine, a known blocker of autophagy. After separately characterizing LAMP1 lysosomes (Figure 1B) and LC3^^+^^ autophagosomes (Figure 2A), double labeling demonstrated that swollen lysosomes fused with autophagosomes and double-positive compartments significantly increased by 1-TbAd treatment (*P* < 0.0001) (Figure 2B). Next, we tested the effect of 1-TbAd and *N*^^6^^-TbAd on autophagosome numbers in mouse RAW264.7 cells. We observed an increase in the number and intensity of LC3B-II^^+^^ puncta in response to 1-TbAd treatment, again demonstrating an accumulation of autophagosomes. Even at this relatively early 4-hour time point, some of these LC3BII^^+^^ autophagosomes clearly colocalized with LAMP1, often in rings around LAMP1^^+^^ puncta (Figure 2C).

### 1-TbAd blocks autophagic flux.

Accumulation of LC3^^+^^ vesicles (Figure 2, A–C) could result from a blockade of lysosome-mediated degradation, as hypothesized, or increased autophagosome biogenesis. To distinguish these outcomes, we measured autophagic flux with the GFP-mCherry-LC3B reporter system. Whereas mCherry is stable at acidic pH, GFP fluorescence is quenched, so mature autophagolysosomes with low pH appear red, and alkalinized autophagosomes appear yellow (46). In vehicle-treated RAW264.7 macrophages, most mCherry^^+^^ puncta were GFP^^–^^, suggesting efficient autophagosome-lysosome fusion, low pH, and a degradative microenvironment (Figure 2D). Starvation-induced autophagy did not result in an increase in GFP puncta per cell and only marginally increased the intensity of GFP signals in mCherry puncta, suggesting the expected rapid fusion of autophagosomes with lysosomes to degrade their content. Bafilomycin A1 (BafA1) inhibited v-ATPase function, leading to increased GFP puncta and fluorescence intensity, along with nearly complete colocalization with mCherry, indicating a buildup of immature, pH-neutralized autophagosomes (Figure 2D).

Macrophages treated with 1-TbAd, but not *N*^^6^^-TbAd, showed 2 clear effects. Cells accumulated GFP and mCherry double-positive structures, providing direct evidence for 1-TbAd–induced failure in autophagosomal acidification (Figure 2D). Second, autophagosomes appeared swollen, with ring-shaped LC3BII^^+^^ limiting membranes (Figure 2D), whereas BafA1-induced yellow compartments remained punctate. BafA1 generated more puncta per cell than did 1-TbAd, but lower GFP intensity. Thus, BafA1 and 1-TbAd both strongly inhibited acid quenching of the GFP fluorescence, but only 1-TbAd caused dilation of autophagosomes. These distinct outcomes likely reflect the separate mechanisms of BafA1 in proton pump inhibition versus 1-TbAd–induced lysosomotropic entry and swelling. These clear outcomes directly link 1-TbAd action to lysosomal swelling, alkalinization, and accumulation of immature autophagosomes.

### TbAd induces protein and lipid autophagic cargo.

To test whether 1-TbAd also causes a buildup of autophagocytic cargo, we detected autophagy markers in Western blotting of RAW264.7 mouse macrophages treated with 1-TbAd. With short (2 h) and long (4 h) treatment durations in 3 experiments, we found that 1-TbAd, chloroquine, and BafA1 all induced the membrane marker LC3-II and p62 protein cargo of autophagosomes (Figure 2E). The proposed lysosomotropic mechanism requires antecedent acidification of lysosomes, where the lysosomal pH gradient drives 1-TbAd entry, followed by partial dissipation of the pH gradient. In agreement with this model, further experiments showed that autophagic cargo accumulation was not additive for BafA1 plus 1-TbAd (Supplemental Figure 5). This observation can be explained if BafA1 dissipates the pH gradient needed for 1-TbAd to penetrate lysosomes, or if BafA1 effects are maximal. Overall, 1-TbAd induced autophagic cargo accumulation, reinforcing the idea that increased LC3-II was likely due to decreased autophagosome degradation, not increased biogenesis.

### Lipidomic analysis of 1-TbAd–induced lipid storage.

**Figure 3:**
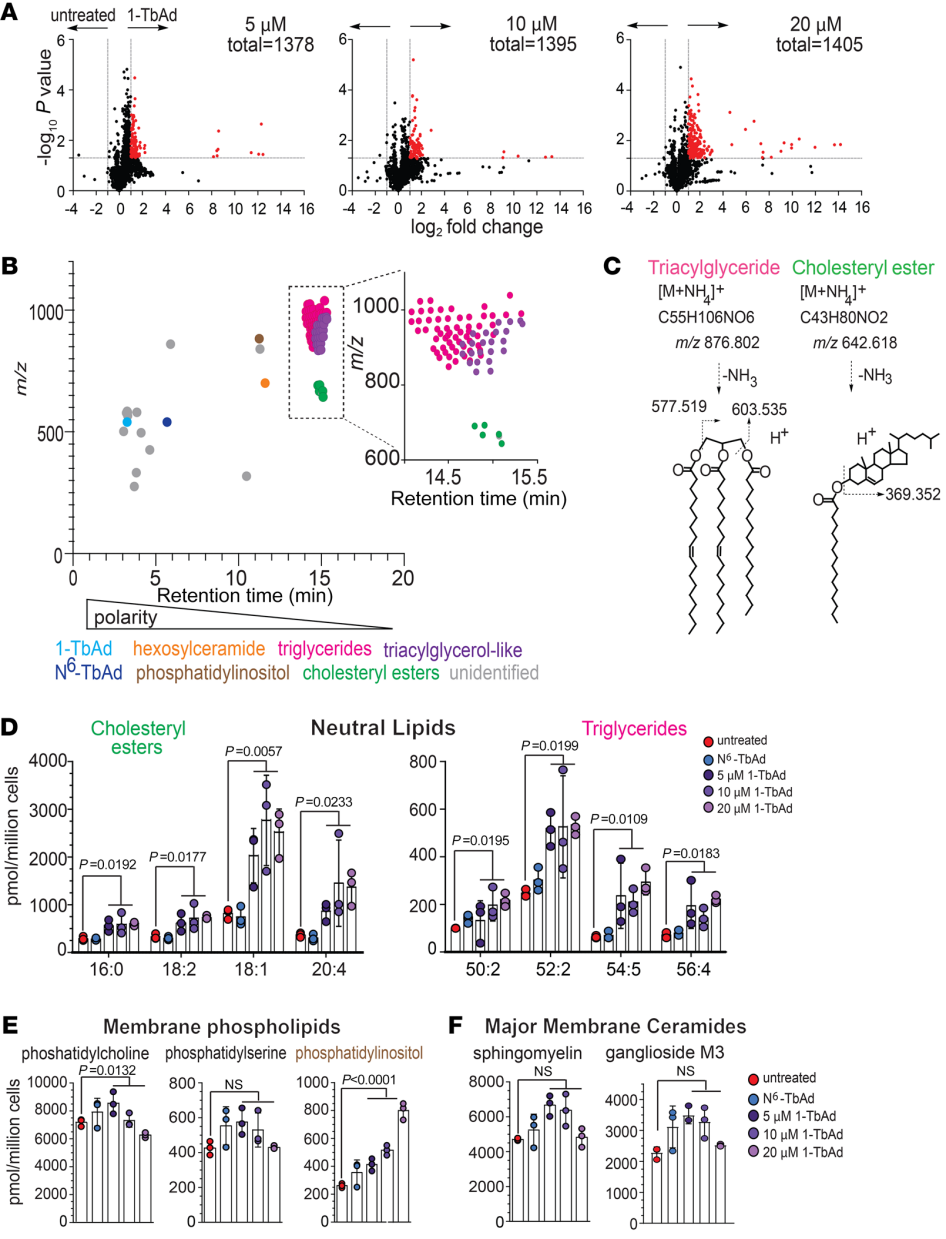
Lipidomic analysis of 1-TbAd–induced lipid storage in macrophages. (**A**) Human macrophages were treated in biological triplicate and normalized to the cell number prior to lipid extraction. Positive-mode HPLC-MS lipidomics data sets were aligned, and intensity ratios for every detected lipid allowed the identification of changed molecular events (red, *P* < 0.05, >2-fold). (**B**) Unique changed molecular events were plotted by retention time and *m/z*, where structurally related molecules cluster. (**C**) Lead ions in TAG and CE clusters were identified on the basis of the mass of ammonium adducts and the diagnostic cleavage. (**D**–**F**) The quantities of PC, PS, PI, and sphingomyelin (SM) were assigned on the basis of authentic standard curves (Supplemental Figure 7). The GM3 structure was solved by CID-MS and coelution with an authentic standard (Supplemental Figure 8). *P* values in **D**–**F** were determined by 1-way ANOVA followed by a post test for linear trend.

To broadly measure lipid changes in macrophages, we used an in-house–developed lipidomics platform to measure all ionizable lipids as “molecular events” (47). Macrophages were treated with 0, 5, 10, or 20 μM doses of 1-TbAd (Figure 3A) to detect 1,378, 1,395, and 1,405 molecular events, respectively, demonstrating the breadth and reproducibility of lipidomic detection. We aligned treated and untreated cellular data sets to assess the gain or loss of signal for every ionizable lipid and identified the lipids meeting the change criteria (2-fold and corrected *P* < 0.05) (Figure 3A, red). Overall, greater than 99% of changed lipids were upregulated by 1-TbAd, leading to highly asymmetric volcano plots (Figure 3A). Like BODIPY staining (Figure 1, E and F), this result again documented marked lipid overload, and the effects were dose responsive to 1-TbAd.

Only 9%–13% of lipids met the change criteria, making clear that only certain subclasses of cellular lipids were affected. A separate study of changed and unchanged events allowed the identification of cellular patterns of lipidic change. In addition to added 1-TbAd and its *N^6^*-TbAd rearrangement product (27, 28) (Figure 3B, blue), we detected approximately 200 changed events corresponding to host lipids in the lipidome, so we implemented strategies to identify lipids in groups (48). Among 1,405 total events and 193 changed events, we detected 105 unique events after filtering isotopes and alternate adducts, which were plotted on *m/z* and retention axes to reveal a clustering pattern (Figure 3B). Embedded accurate mass data were used to identify 1 lipid in each cluster. For example, *m/z* 876.802 matched the ammonium adduct of TAG with 52 C and 2 unsaturations (52:2 TAG). Collision-induced dissociation (CID) mass spectrometry (MS) detected diacylglycerols (*m/z* 577.519 and 603.535), ruling in TAG structure (Figure 3C). The 61 nearly coeluting ion chromatograms (Figure 3B, pink) differed by mass intervals matching CH2 (*m/z* 14.015 amu) or H2 (*m/z* 2.015 amu) to describe chain length and saturation variants (Supplemental Figure 6). Another 1-TbAd–induced compound (*m/z* 642.618) with a high fold change matched a cholesteryl ester with a 16:0 fatty acyl unit (16:0 CE), which yielded a defining dehydrocholesterol (*m/z* 369.352) fragment (Figure 3C). Four additional CEs with distinct fatty acyl moieties comprised the 5-member cluster (Figure 3B, green). By comparing intensity values to external standard curves (Supplemental Figures 7–9), MS signals estimated absolute cellular lipid pool sizes, which were large (>100 pmol/million cells) and showed significant increases in response to 1-TbAd treatment (Figure 3D).

Analysis of lipid pools unaffected by 1-TbAd helped complete the picture of the cellular response. Among membrane phospholipids, PC (*m/z* 760.585) and phosphatidylserine (PS) (*m/z* 762.528) were unchanged (Supplemental Figure 8 and Figure 3, E and F). Only phosphatidylinositol (PI), a less abundant lipid with dual roles in membrane formation and signaling (49), was increased after 1-TbAd treatment. The 2 major membrane sphingolipids, identified as sphingomyelin and ganglioside M3 (GM3) by HPLC-MS (Supplemental Figure 9), were unaffected by 1-TbAd (Figure 3F). Thus, lipid storage patterns did not reflect a global lipid anabolic state, but instead selectively involved certain neutral lipids, especially TAGs and CEs. Notably, the lipidomic pattern matched the expected outcome of lysosomal hydrolases, as documented in our microscopy experiments (Figure 1, B and C, and Figure 2, A–D). For example, CE catabolism occurs in lysosomes, and CEs accumulate with even relative deficiencies of LAL or LAL inactivation by chloroquine (17).

### 1-TbAd phenocopies human lysosomal storage diseases.

**Figure 4:**
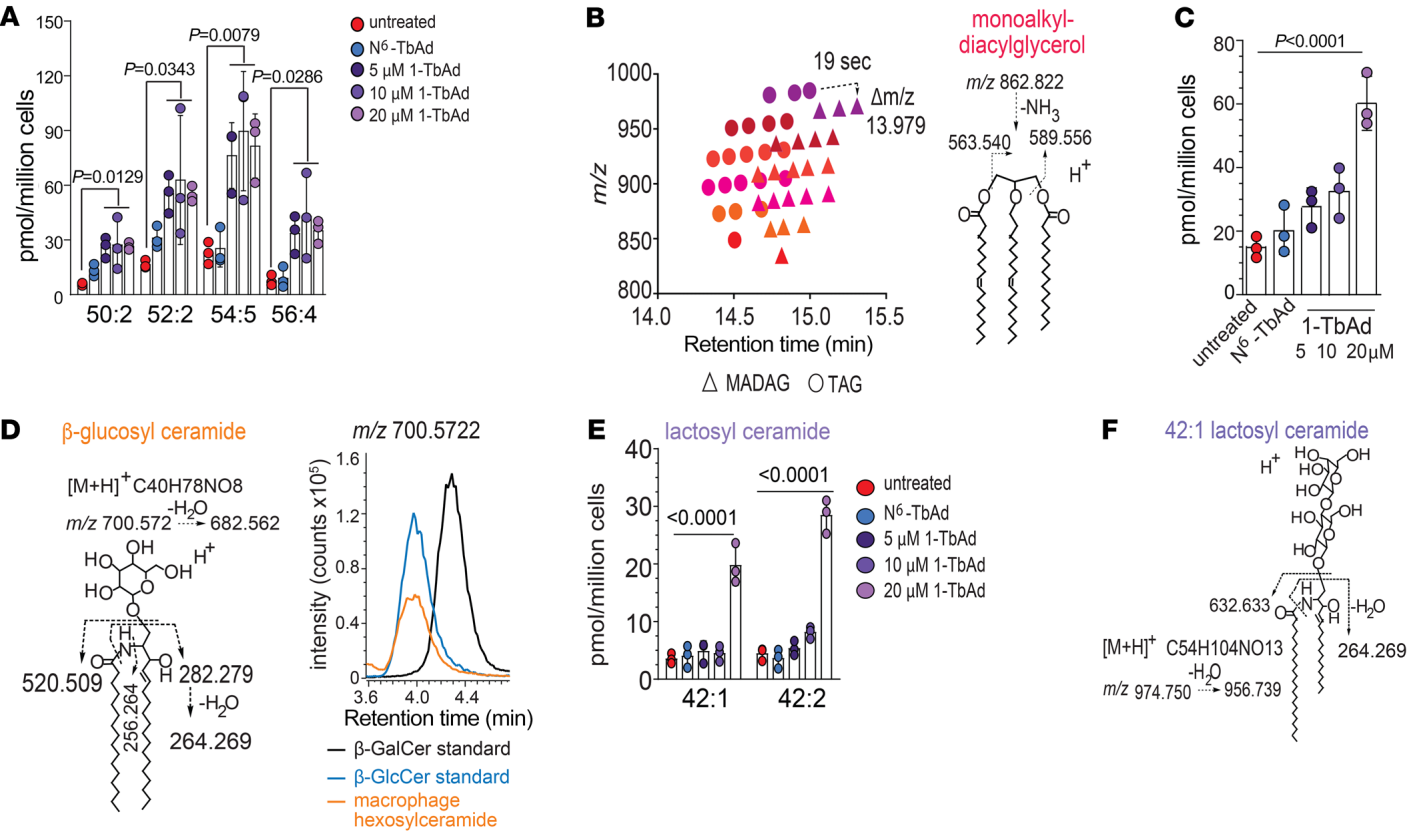
1-TbAd induces storage of known substrates in lysosomal storage diseases. (**A**) Unknown lipids could be linked on a 1-to-1 basis with TAGs (dashed arrow) based on retention time (~19 s) and mass (13.979 mu) increments, which correspond to an ether linkage substituting an ester linkage, suggesting that the unknowns were MADAGs. (**B**) The MADAG structure was confirmed by CID-MS. (**C**) After quantitation using TAG as the external standard, the dose response to 1-TbAd of 4 MADAGs with the indicated chain length and saturation pattern was reported. *P* values were determined by 1-way ANOVA followed by post test for linear trend. (**D**) The 1-TbAd–induced hexosylceramide in macrophages matches the structure of β-glucosylceramide, based on CID-MS and coelution with an authentic internal standard. (**E**) A C42:2 dihexosylceramide induced by 1-TbAd was solved as LacCer, based on CID-MS and coelution with an authentic standard. The *P* values for **A**, **C**, and **E** were determined by 1-way ANOVA followed by Dunnett’s multiple-comparison test.

Next, we identified a large family of TAG-like unknowns induced by 1-TbAd (Figure 3B, purple). The masses of the most abundant compound (C59H104O5) and its 20 variants did not match the database searches. This puzzle was solved by observing that each unknown eluted approximately 19 seconds later and showed 13.979 lower *m/z* values compared with TAGs. The apparent loss of O and gain of 2H (Figure 4A) suggested that an ether linkage replaced an ester in monoalkyldiacylglycerides (MADAGs) (50) (Figure3B and Figure 4A). Confirming this hypothesis, CID-MS of *m/z* 862.822 identified monoalkylmonoacylglycerol fragments (*m/z* 563.540, 589.556), and the cleavage-resistant ether bond (Figure 4B). The effect of 1-TbAd on MADAGs was strong (Figure 4C). MADAGs are notable because they are rarely reported in normal cells, but are the defining stored substrate in Wolman’s disease (51), which results from inactivation of LAL, the same lysosomal hydrolase controlling TAG and CE breakdown (17, 50). Thus, 1-TbAd treatment biochemically phenocopied Wolman’s storage disease in its 3 major lipid substrates. There was no basis for us to expect specific inhibition of LAL, but 1-TbAd did raise lysosomal pH (Figure 2D), which is expected to allosterically inactivate LAL, along with approximately 60 lysosomal hydrolases (2). Therefore, such 1-TbAd–induced pan-lysosomal failure is predicted to generate additional storage substrates of pH-regulated enzymes.

**Figure 5:**
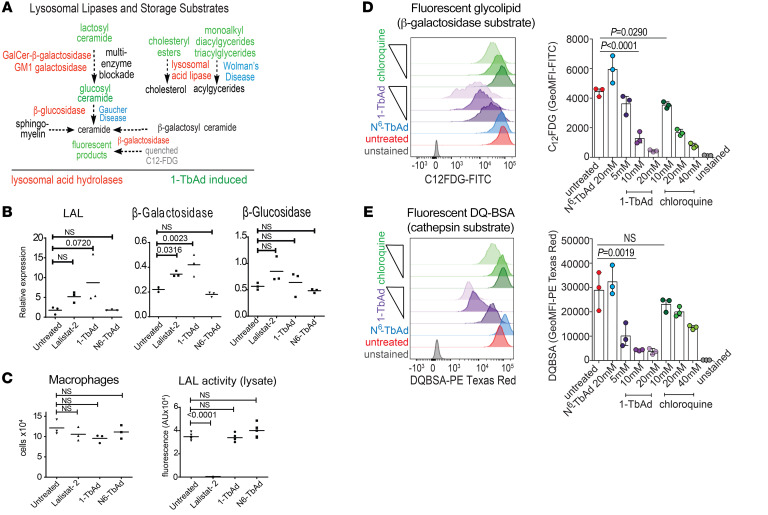
Analysis of 1-TbAd effects on enzymes and substrates known from human lysosomal storage diseases. (**A**) The known relationships among substrates that define human genetic lysosomal storage diseases are indicated (53), emphasizing products that are 1-TbAd induced (green) or involved in eponymous lysosomal storage diseases (blue). (**B**) Human macrophages were treated with 20 μM 1-TbAd for 4 hours and subjected to RT-PCR. (**C**) Human M1 macrophages were treated with TbAd (20 μM) or lalistat-2 (100 μM), counted, and then lysed to fluorometrically measure turnover of P-PMHC as a quantitative measure of LAL action. *P* values were derived from an ordinary 1-way ANOVA with Dunnett’s multiple-comparison test. (**D** and **E**) Human macrophages were pretreated with the indicated compounds, followed by flow cytometric measurement of glycolipid (C_12_FDG) or protein (DQ-BSA) probes. *P* values were determined by the Kruskal-Wallis multiple-comparison test.

Fulfilling this prediction, an unknown lipid (*m/z* 700.56, 11.8 min) showed strong (2.4-fold) and significant (*P* < 0.0001) induction (Figure 4A). CID-MS demonstrated a neutral loss of hexose, leaving ceramide (520.509) and its sphingosine chain (*m/z* 282.279) (Figure 4B). The unknown coeluted in HPLC-MS with β-glucosylceramide (52), not β-galactosyl ceramide, provided as external (Figure 4D) and internal (Supplemental Figure 10) standards. Thus, 1-TbAd induced the defining substrate of Gaucher’s disease, which results from the inactivation of the lysosomal glycoside β-glucosidase (53). Then, we identified another 1-TbAd–induced lipid (Figure 3B) as lactosyl ceramide (LacCer) based on its exact mass, CID-MS fragments, and coelution with a standard (Figure 4, E and F, and Supplemental Figure 9). LacCer is cleaved by either of 2 lysosomal enzymes, so it does not typically accumulate after single gene defects (Figure 5A), but appears after saposin deficiency or other causes of pleiotropic lysosomal glycosidase inactivation (53).

### Transcriptional versus functional enzyme regulation.

An alternate hypothesis is that the effect was due transcriptional downregulation of lysosomal enzymes (Figure 5A). However, 1-TbAd induced no decrease in LAL, β-glucosidase, or β-galactosidase transcripts (Figure 5B). Further, cellular LAL bioactivity on the substrate 4-propyl-8-methyl-7-hydroxycoumarin measured in lysed macrophages was not affected by 1-TbAd, suggesting preserved LAL protein function. Since lalistat-2 completely blocked LAL bioactivity under these conditions, this finding also pointed away from any direct LAL inhibition by 1-TbAd itself (Figure 5C). Instead, the favored pan-lysosomal pH inactivation mechanism whereby 1-TbAd raises pH to cause reversible inhibition is simple, and it is supported by the demonstrated elevation of pH (Figure 2D), the known pH dependence of lysosomal enzymes (2), the lipidomics pattern showing accumulation of 5 known lysosomal storage substrates (Figure 5A), and the localization of stored lipids to lysosomes (Figure 1F).

To directly test this unifying hypothesis in cells, we measured 5-dodecanoylaminofluorescein di-β-D-galactopyranoside-C12 (C__12__FDG), a lysosomal substrate of β-galactosidase (54). C__12__FDG is self-quenched by the conjugated fluorophores, and cleavage induces fluorescence. 1-TbAd–pulsed macrophages showed a dose-dependent reduction in fluorescence. 1-TbAd was more potent than chloroquine, and it showed a strong (~30-fold) and significant effect (*P* < 0.0001) (Figure 5D). The pan-lysosomal hypothesis further predicts that enzyme arrest might extend to proteases, so we assayed DQ-BSA, another self-quenching substrate that is cleaved by cathepsins in endosomes (55). Again, 1-TbAd had a stronger effect than chloroquine, as the former exhibited a dose-responsive, significant (*P =* 0.0019), and strongly (~20-fold) reduced fluorescence (Figure 5E). Overall, 1-TbAd raised the intralysosomal pH and strongly and selectively inhibited acid-dependent glycosidase and protease function within intact cells.

### Tuberculosinyl metabolites during infection.

**Figure 6:**
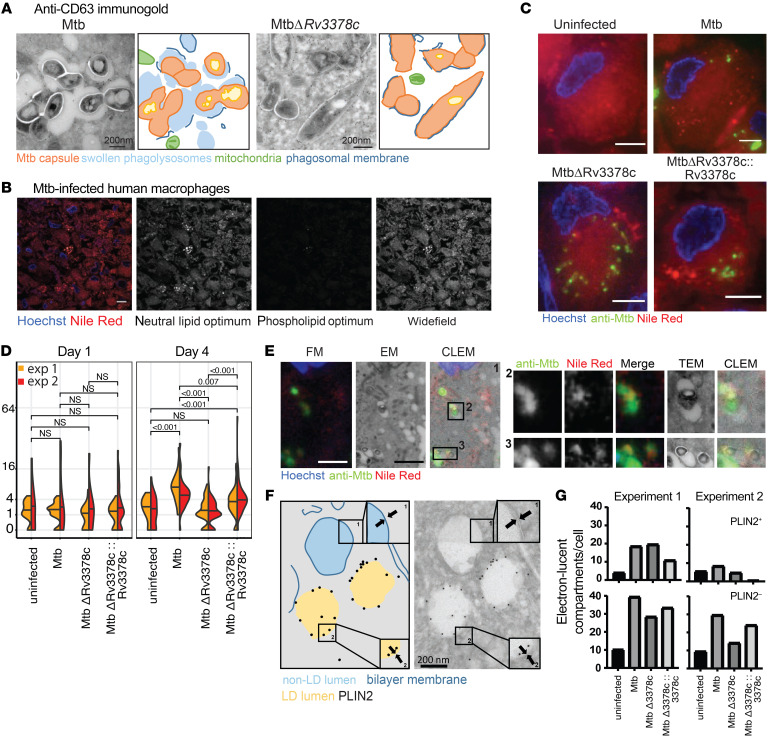
*M. tuberculosis*–produced 1-TbAd induces lipid accumulation in human macrophages. (**A**) Human M1 macrophages were infected with *M. tuberculosis* or MtbΔ*Rv3378c* for 4 days, as reported previously (32), and were then subjected to anti-CD63 staining and annotated. Scale bars: 200 nm. (**B**) In a separate infection with WT *M. tuberculosis*, representative TEM images taken over 4 days showed lysosomal swelling. (**C**–**E**) Immunofluorescence images of human M1 macrophages infected for 4 days were stained with Hoechst (blue), anti–*M. tuberculosis* protein (green), and lipids with Nile red (red). The Nile red images were captured in excitation/emission detection windows that allowed broad detection of lipids (wide-field, 532–538 nm/570 nm), as well as detection of neutral lipids (515 nm/585 nm) and phospholipids (554 nm/638 nm). Wide-field Nile red puncta were quantified in 2 experiments with 35–56 cells for each infection condition. *P* values in panel **D** were determined by a least-squares means post test with adjustment by Tukey’s method after fitting a generalized linear mixed model and factorial ANOVA (overall *P* < 0.001 for strain). Data from 2 experiments were pooled after determining that the model fit was unchanged. In panel **E**, CLEM analysis of human macrophages infected for 4 days identified infected compartments and the limiting membranes of infected phagosomes with visible bacilli, along with staining for lipids (Nile red) and anti–*M. tuberculosis* antisera. Scale bars: 5 μm (**B**, **C**, and **E**). FM, fluorescence microscopy. (**F** and **G**) Human macrophages were infected with *M. tuberculosis* for 4 days, followed by staining with anti-PLIN2 immunogold. High-magnification images (insets 1 and 2) show a membrane bilayer and monolayer, respectively. In 2 independent experiments, 3,661 electron-lucent compartments stained with (PLIN2^+^) and without (PLIN2^–^) immunogold were counted in 9–17 cells per condition.

*M. tuberculosis* H37Rv produces approximately 6 ng/10^^8^^ bacteria of 1-TbAd, suggesting that 1-TbAd could plausibly accumulate to low micromolar concentrations during macrophage infection (32). Therefore, we asked if responses to synthetic 1-TbAd resemble the differences in the macrophage response to infection by the 1-TbAd–producing *M. tuberculosis* strain H37Rv versus the tuberculosinyl transferase–deficient mutant (MtbΔ*3378c*). Extending prior EM analyses (32), we performed immunogold staining of M1 macrophages at day 4 of infection and observed markedly swollen phagolysosomes in comparison with 1-TbAd–deficient MtbΔ*3378c* (Figure 6A). These studies also detected abundant immunogold staining of a lysosomal marker (CD63) in infected compartments, suggesting that phagosome-lysosome fusion occurred in the presence of 1-TbAd. Like 1-TbAd–treated cells (Figure 1, B and C, and Figure 2B), the swollen CD63^^+^^ phagosome-lysosome space contained particulates, suggesting peribacillary lipid storage. Mitochondria, as an example of nonacidified organelles (Figure 6A, green), lacked swelling and inclusions. Although the Rv3378c-dependent component of live infection phenocopied the lysosomal swelling seen in response to pure 1-TbAd, the infectious process occurred more slowly, whereby the mutant first diverged from WT *M. tuberculosis* at day 4 of infection (Supplemental Figure 11), rather than at 4 hours (Figure 1, A and B).

Both pure 1-TbAd (Figure 1) and a 4-day *M. tuberculosis* infection (Figure 6, B and C) induced punctate Nile red staining patterns. Lysosomal storage diseases more strongly affect neutral lipids than phospholipids, as the former accumulate as cargo inside membrane-bound compartments (53). Using fluorescence emission windows to optimize neutral lipid and phospholipid detection, we found that *M. tuberculosis*–induced lipid inclusions had stronger neutral lipid signals (Figure 6B), matching the neutral lipid patterns seen in lipidomics (Figures 3 and 4).

Uninfected cells showed diffuse Nile red staining corresponding to membranes, but *M. tuberculosis*–infected cells showed puncta that were lipid inclusions. Quantitation of puncta across 2 experiments at 1 day showed an increase in 1-TbAd–replete strains, as compared with uninfected cells and the MtbΔRv3378c strain. However, this weak effect was present only in 1 experiment and was not statistically significant. In both experiments, by 96 hours, we observed highly significant (*P* < 0.007) induction of lipid inclusions for WT and H37Rv-complemented bacteria as compared with uninfected cells and the MtbΔRv3378c strain (Figure 6D). Anti–*M. tuberculosis* antisera helped us to assess the degree of infection as a covariate and showed that individual cells with high infection could have low lipid inclusions when 1-TbAd was not produced (Figure 6C). Finally, similar to the effects of pure 1-TbAd (Figure 1F), CLEM revealed that *M. tuberculosis*–infected cells localized lipid inclusions to LAMP1^^+^^ compartments (Figure 6E). The presence of bacteria provided a reference to localize lipid inclusions to the peribacillary space of infected phagolysosomes as well as compartments without visible bacteria. Overall, higher lysosomal swelling and lipid accumulation occurred in response to 1-TbAd–producing *M. tuberculosis* strains, which phenocopied 1-TbAd treatment, with the key exception that infection-induced events unfolded over several days.

### Lipid droplets after 1-TbAd treatment.

Intralysosomal lipid storage is the expected proximate outcome of inactivation of hydrolases located in phagolysosomes. However, lipid inclusions also occur in lipid droplets (LDs), which are specialized perilipin^^+^^ organelles with a unique single-layer membrane (56). Immunogold EM staining colocalized perilipin 2 (PLIN2) and the highly characteristic single lipid layer, as contrasted with the lipid bilayer of lysosomes (Figure 6F, insets). After a 4-day infection with WT H37Rv, Rv3378c-mutant strains, or Rv3378c-complemented strains, macrophages lacked consistent changes in the appearance or number of PLIN2^^+^^ LDs (Figure 6, F and G). However, PLIN2^^–^^ electron-lucent compartments fortuitously showed an observable increase in *M. tuberculosis*–infected cells, which correlated with the 1-TbAd status of the strains and reached significance in 1 experiment, suggesting non-LD lipid accumulation (Figure 6G). Although we cannot rule out secondary roles of LDs, immunofluorescence (Figure 1A), EM (Figure 1B, Figure 2, A and B, and Figure 6A), and CLEM (Figure 1C) all identified lipid storage in LAMP1^^+^^, CD63^^+^^, or visibly infected phagolysosomal compartments.

### 1-TbAd promotes M. tuberculosis growth in macrophages.

**Figure 7:**
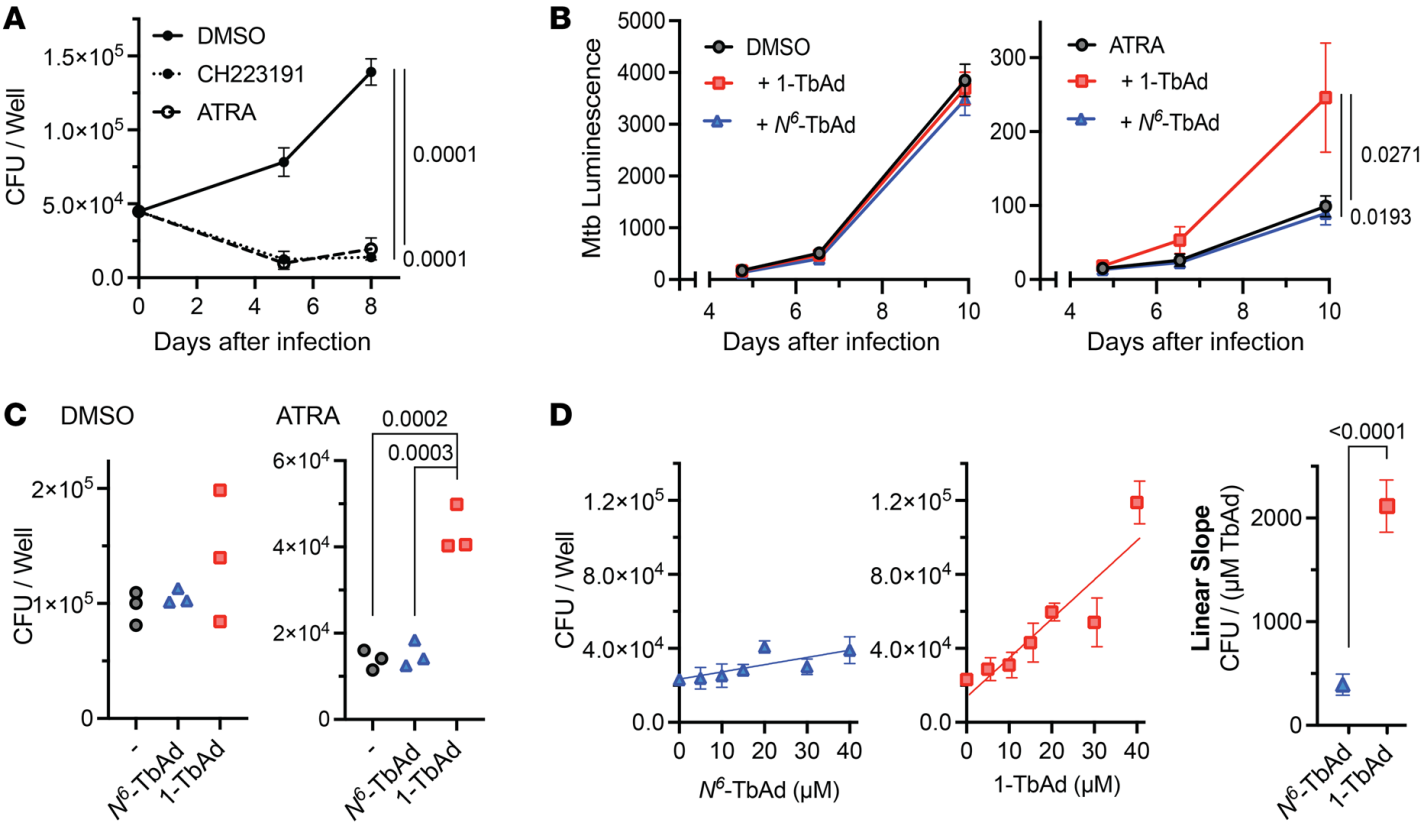
1-TbAd reduces macrophage control of *M. tuberculosis*. (**A**–**C**) Mouse BMDMs were infected with *M. tuberculosis* (MOI = 2) for approximately 6 hours, pulsed with TbAd for 3 hours, and then treated with ATRA (10 μM), CH223191 (3 μM), or DMSO. Macrophages were infected with *M. tuberculosis*, with or without a 20 μM TbAd pulse, followed by measurement of CFU (**A**) bacterial luminescence reporters (**B** and **C**) for 10 days (**C**) or 7 days (**B**). (**D**) Macrophages were infected with WT *M. tuberculosis*, pulsed with TbAd, and treated with ATRA (10 μM) for 6 days prior to CFU measurement. Statistical comparisons in **A**–**C**)were performed using an ordinary 1-way ANOVA with Tukey’s or Šídák’s multiple-comparison test (all comparisons tested, *P* values are shown where *P* < 0.05). Comparisons with multiple time points (**A** and **B**) were performed on AUC data. Statistical comparison of slopes in **D** was performed using an unpaired *t* test.

1-TbAd–induced lipid accumulation may allow bacterial access to cholesterol or other favored lipid carbon sources to support *M. tuberculosis* growth (25). However, survival factors are penetrant only under conditions of growth control. In some circumstances, mouse macrophages show poor *M. tuberculosis* control, but all-*trans*-retinoic acid (ATRA) limits growth by restricting access to cholesterol (24, 57). We confirmed that ATRA severely restricted *M. tuberculosis* growth in murine bone marrow–derived macrophages (BMDMs), similar to CH223191, a candidate for macrophage-directed *M. tuberculosis* therapy via the aryl hydrocarbon receptor (58) (Figure 7A). This restriction was abrogated by 1-TbAd treatment, when *M. tuberculosis* growth was measured by a bacterial luminescence reporter (Figure 7B) and confirmed by CFU measurements (Figure 7C), with separate experiments revealing a significant dose-dependent effect specific to the lysosomotropic 1-TbAd isomer (Figure 7D). Notably, no such impact of 1-TbAd on *M. tuberculosis* intramacrophage growth was observed in macrophages that were not treated with ATRA (Figure 7, B and C). This finding demonstrates a survival effect of 1-TbAd and links it to the ATRA-induced nutritional status of the macrophage, providing another link to suggest that 1-TbAd acts by allowing bacterial access to nutrients.

### Pharmacologic reversal of 1-TbAd lysosomal dysfunction.

**Figure 8:**
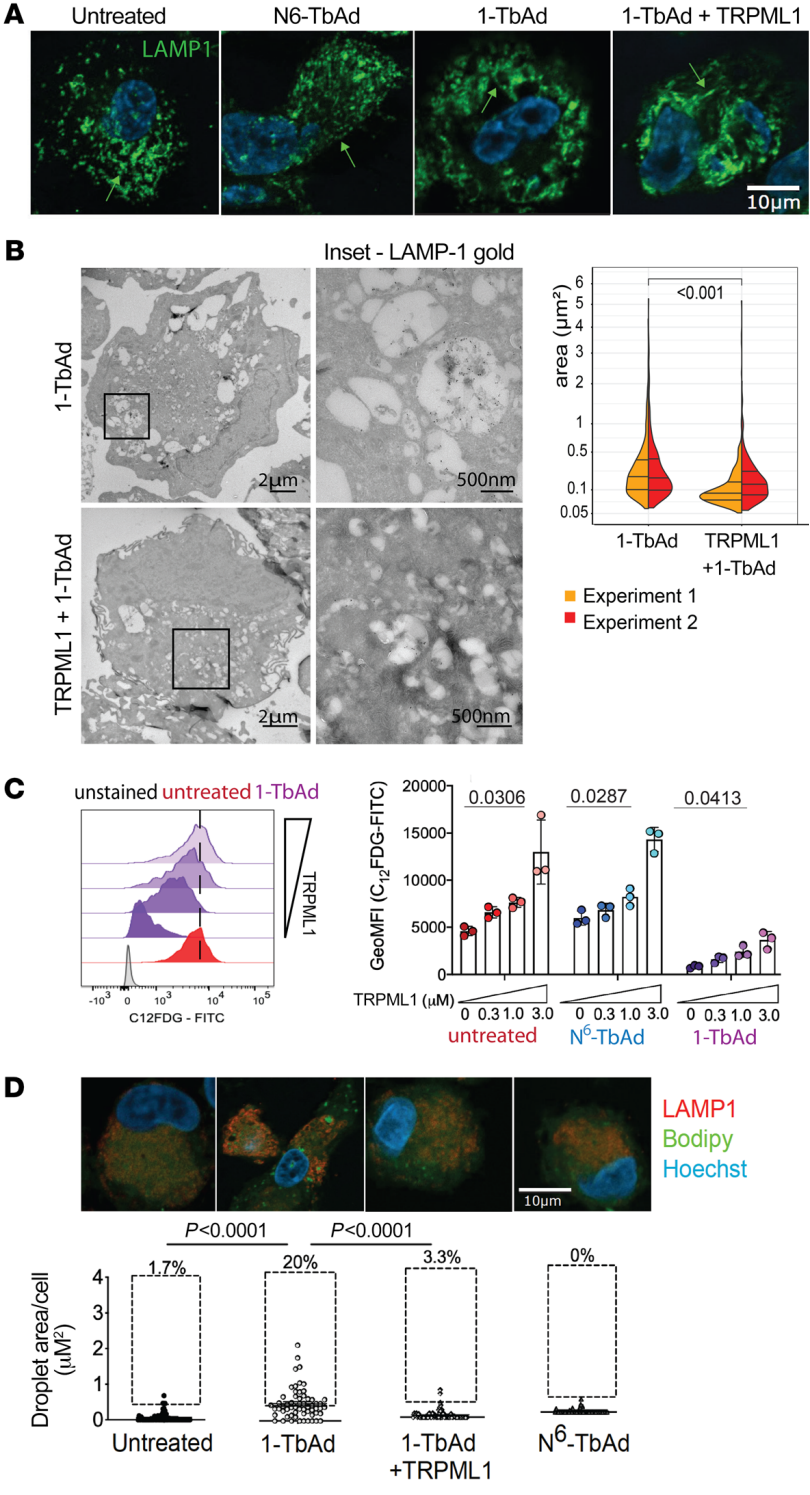
TRPML1 agonism prevents 1-TbAd effects on macrophages. (**A**) Human M1 macrophages were pretreated with TRPML1 agonist for 1 hour, followed by incubation with 10 μM 1-TbAd for 4 hours. Scale bar: 10 μm. (**B**) Cells treated as in **A** were labeled with 10 nm immunogold and anti-LAMP1. Insets of representative images show electron-lucent compartment inclusions (upper right), while agonist-treated macrophages contained smaller electron-lucent compartments (lower right). The area (μm^2^) of electron-lucent lysosomal compartments was determined using the least-squares mean post test with adjustment by Tukey’s method after factorial ANOVA of a linear mixed model fit that treated each cell as a random effect variable. Scale bars: 2 μm and 500 nm (enlarged insets). (**C**) Human M1 macrophages loaded with the C_12_FDG were treated as in **A** in biological triplicate in 2 experiments. (**D**) Human M1 macrophages treated as in **A** were subjected to BODIPY and anti-LAMP1 staining followed by BODIPY quantitation with the Kruskal-Wallis test. The results are representative of 3 experiments. Scale bar: 10 μm.

To further test causal links of 1-TbAd with lysosomal function and to investigate the feasibility of therapeutic reversal, we treated macrophages with C8, a selective agonist of the transient receptor potential mucolipin 1 (TRPML1) channel (Figure 5A). TRPML1 activates v-ATPases to lower lysosomal pH and broadly increase enzymatic catabolism (59, 60) and lyososomal Ca^^2+^^ egress, which drives the transcription of lysosomal proteins (61) and autophagosome biogenesis (62). We analyzed human macrophages that were or were not pretreated with C8 for 1 hour prior to a 1-TbAd pulse. In contrast to 1-TbAd induction of large lysosomes, the LAMP1^^+^^ compartments of C8-treated cells largely remained in a punctate form, similar to the untreated and *N*^^6^^-TbAd–treated cells (Figure 8A). Also, lysosomes in the C8-treated cells became tubulated (Figure 8A, arrow), an expected effect seen in lysosome biogenesis (63). Immunogold EM analysis with anti-LAMP1 showed that C8 reduced the size of lysosomes by more than 2-fold (*P* < 0.001) in 1-TbAd–pulsed human M1 macrophages (Figure 8B).

The C__12__FDG assay showed that C8 provided a strong (~7-fold) effect to reverse the blockade of glycolipid catalysis of 1-TbAd–treated cells (Figure 8C). Similarly, immunofluorescence analysis of BODIPY-stained lipid inclusions showed significantly (*P* < 0.0001) reduced lipid inclusions (Figure 8D). In C12FDG catalysis assays and BODIPY storage assays, agonist-treated cells showed outcomes that were similar to those seen with cells not treated with 1-TbAd, indicating robust protection. In all 4 types of experiments, agonist effects could be specifically linked to lysosomes by defined criteria, including lysosome-specific localization of TRPML1 channels (64), the known catabolism of C__12__FDG in lysosomes, the LAMP1^^+^^ tubulation phenotype, and expression of LAMP1^^+^^ by the swollen compartments. These data provide a potential point of entry for host-focused “lysosomal” therapy, since key effects of 1-TbAd on human macrophages can be largely prevented by a drug that acts on upstream control pathways to broadly restore lysosomal homeostasis.

## Discussion

Christian De Duve coined the term “autophagy” and demonstrated the essential role of lysosomes in degrading autophagic cargo using the pharmacological lysosomotrope chloroquine (31). The effects of 1-TbAd on host macrophages fulfill the predictions of the lysosomotropic model regarding delayed timing, durability after pulse, and lysosome specificity. We observed lysosomal swelling among all cell types tested, fulfilling another prediction of lysosomotropism: it is a mass action–driven chemical effect that should occur in any cell type with acidic lysosomes and does not depend on cell-specific pores or receptors. The pulse-chase model helps establish the sequence of events: swelling is accompanied in time by pH elevation, protease inhibition, and lipase inactivation within 4 hours. A second phase of lipid accumulation occurred over days, where small lipid bodies fused to form massive (>1 μM) inclusions in lysosomes. Both phases occurred during live infection, but they unfolded more slowly over days due to shedding of 1-TbAd from a few bacteria per cell. During infection, the loss of effect with tuberculosinyl transferase deletion specifically implicated terpenyl metabolites as the specific cause of lysosomal swelling and lipid accumulation.

We highlight the antacid role of 1-TbAd because acid pH is the central upstream regulator of approximately 50 pH-dependent, lysosomally localized hydrolases (2). The unbiased lipidomics screen revealed patterns of lipid storage substrates that matched the predictions of lysosomal failure, and neutral lipids were localized as punctate inclusions in membrane-bound lysosomal compartments. Lipidomics and fluorescence substrate assays also provided independent linkages to known substrates of lysosomal storage diseases, including hydrolases, CEs, TAGs, MADAGs, lactosyl ceramide, β-galactosyl ceramide, and β-glucosyl ceramide. Further, 1-TbAd was sufficient to raise intralysosomal pH and inhibit autophagic flux, which are 2 cellular outcomes of infection by *M. tuberculosis* (12, 13). The strong accumulation of LC3-II^^+^^ membranes and p62 indicates that 1-TbAd arrest occurred after the stage of lysosome-autophagosome fusion.

Unlike M1 macrophages, the effects were weaker in AM and M2 macrophages, but were not interpreted as general resistance to 1-TbAd action. Instead, lysosome inactivation was predicted to be a universal cellular response to lysosomotropes and was seen here in all cells tested (31), whereas lipid storage is a secondary cell type–specific effect that depends on the balance of lipid uptake, anabolism, and catabolism. Higher lipid storage in M1 macrophages might be explained if they have lower baseline expression of lipid catabolic genes as reported recently (42, 43). Whereas this study focused on the downstream effects of lysosomal failure on glycolipids, proteases, and autophagy substrates, future work may focus on other TB disease–relevant effects related to lysosomal failure (2). These effects include acid- and cathepsin-dependent MHC II antigen–processing reactions (44) and direct pH-dependent intracellular killing mechanisms (65).

Mycobacteria are sterol parasites that have dedicated catabolic pathways for branched lipids, and both cholesterol (24, 57) and TAGs support mycobacterial growth and persistence in vivo (66–68). Growth-suppressive effects of ATRA can reversed by cholesterol (24) or 1-TbAd. Therefore, 1-TbAd induction of cholesteryl metabolite storage is consistent with the interpretation that the 1-TbAd–induced survival occurred through increased access to lipids. While ATRA has other effects on cells, the favored lipid storage hypothesis is independently supported by observed 1-TbAd–induced lipid inclusions in PLIN2^^–^^LAMP1^^+^^ lysosomes and infected phagolysosomes, which could provide access to the cholesteryl esters, TAGs, lactosyl ceramides, hexosyl ceramides, and other accumulated lipids detected here.

Inborn lysosomal storage diseases provide an analogy to understand mycobacteria-induced lipid storage in TB lesions (69–71). TAGs, MADAGs, β-glucosylceramide, and lactosylceramide accumulation demonstrates that 1-TbAd exposure biochemically phenocopies human genetic deficiencies underlying Wolman’s disease, Gaucher’s disease, and polygenic lysosomal storage diseases, respectively (53). 1-TbAd does not alter the transcription of key enzymes, and it would not be expected to interact with the active site of diverse enzymes that process lipid and peptide substrates. Instead, we propose a simple model, in which an antacid raises the lysosomal pH, leading to pleiotropic inactivation of lysosomal hydrolases. We know of no precedent for a molecule or gene selectively produced by disease-causing mycobacteria that is necessary for lysosomal failure and foamy macrophage formation.

Considering therapy, the identification of multiple storage substrates supports interventions that target upstream mechanisms to broadly activate lysosomes rather than individual lipases. C8 activates TRPML1 and prevents lysosomal dyshomeostasis in a general way through efflux of calcium stores, which might counter the buildup of an intralumenal positive charge caused by lysosomal accumulation of 1-TbAd, and C8 promotes acidification via vATPases (72). TRPML1 activation promotes the translocation of transcription factor EB (TFEB), which is a master regulator of genes involved in lysosome and autophagosome biogenesis (73). The TRPML1 agonist improves catalytic function and reverses cellular lipid storage phenotypes, and it acts upstream of lysosomal swelling events, creating a tubulation phenotype that suggests lysosomal biogenesis. Our data extend the findings of recent studies demonstrating that TRPML1 agonists correct pathogen-induced lysosomal vacuolization by *Helicobacter pylori* (74), providing a specific point of entry for the development of host-directed TB therapy through lysosome activation.

Finally, the cellular data can guide future work to discover the functions of shed 1-TbAd in TB disease. Tuberculosinyl metabolites are selectively expressed in disease-causing species in the *M. tuberculosis* complex, and they generate lipid storage phenotypes that mimic those seen in TB disease (18, 21). Both our data and prior studies (29) link *Rv3378c* to survival in bone marrow macrophages, and *M*. *kansasii* persistence is improved in alveolar macrophages after TbAd biosynthetic gene transfer (75). However, any essential effects on TB disease are not yet fully defined with regard to host species, time frame of infection, cell type, or organ location. For successful clinical translation, we need to understand how Rv3378c acts in escape from host killing or promoting transmission. The cellular data reported here support this translational process by pointing to immune M1 macrophages that can limit *M. tuberculosis* growth by chronically restricting access to cholesterol and related lipids (24). Also, these data support the feasibility of host-directed therapy aimed at lysosomal activation to augment conventional antimicrobial drugs.

## Methods

### Macrophage culture.

CD14^^+^^ monocytes were isolated with Miltenyi magnetic activated column sorting (MACS) by plating 1 × 10^^5^^ cells/mL in 48 mL for culturing in RPMI-1640 supplemented with 10% FCS (Gibco, Thermo Fisher Scientific), 5 mL penicillin/streptomycin, 5 mL HEPES, 3 mL essential amino acids, 3 mL nonessential amino acids, 2 mL glutamine, and 0.5 mL mercaptoethanol. A total of 1 × 10^^5^^ cells/well in 48-well plates or 1.5 × 10^^7^^ cells in 100 mm dishes were differentiated into M1 macrophages using 25 ng/mL MCSF and 2.5 ng/mL GM-CSF (Peprotech, BioLegend) over 6 days. Cells were lifted with Accutase (Innovative Cell Technologies) and replated in glass-bottomed, 96-well plates (Greiner) for microscopy. For M2 macrophages, GM-CSF was omitted. Expression of CD14, CD16, CD163, CD206, CD80, and HLA-DR (BioLegend) on day 6 was determined by flow cytometry.

Human AMs were retrieved by bronchial washes (76) filtered through 100 μm nylon (BD Falcon) and centrifuged (~150*g*) for 15 minutes. A suspension of 5 × 10^^5^^ cells/mL in RPMI 1640 with 10% FCS (Gibco, Thermo Fisher Scientific), 50 μg/mL amphotericin B, and 50 μg/mL cefotaxime was subjected to adherence purification in 8-well Labtek chamber slides (Nunc) or 24-well plates containing glass coverslips.

RAW264.7 macrophages were cultured in DMEM (Gibco, Thermo Fisher Scientific) supplemented with 10% FCS. Synthetic 1-TbAd and *N^6^*-TbAd were synthesized (37), tested (32), and dried under nitrogen gas in glass tubes and dispersed into media via 60-second water bath sonication or DMSO solvation. In selected assays, cells were pulsed with lipid for 2 hours and washed in media and chased. Oleic acid–BSA and chloroquine (MilliporeSigma) generated lipid overload. TRPML1 agonists C8 (US patent application WO2021/127337A10) were used at 1 μM for 1 hour as described previously (61).

Murine BMDMs from 6- to 8-week-old female C57BL/6 mice were differentiated for 7 days in RPMI-1640 with 10% FCS, 1% GlutaMAX (Gibco, Thermo Fisher Scientific), 10 mM HEPES, 1% antibiotic-antimycotic (Gibco, Thermo Fisher Scientific), and 25% L929-conditioned media. PBS-washed cells were lifted with 2 mM EDTA in PBS and replated in the same media containing only 10% L929-conditioned media.

### Luminescence measurement and CFU enumeration.

Murine BMDMs were seeded at 40,000 cells per well in 96-well plates using clear plastic for CFU and white plastic for luminescence. Cells were infected with the H37Rv or H37Rv-lux strains of *M. tuberculosis* at 2 bacteria per macrophage for approximately 6 hours. After washing with PBS and incubating in media with TbAd for 3 hours, cells were washed again, and media were added with either 0.05% DMSO or 10 μM ATRA. Luminescence (BioTek Synergy H1 microplate reader) was normalized to an untreated well at the final time point. After 6–7 days, CFU in dissociated and adherent fractions were harvested, treated with 0.1% Triton X-100 in media or PBS to achieve complete macrophage lysis, and then diluted in PBS with 0.05% Tween-80 and plated separately onto 7H11 agar in triplicate over 11–16 days.

### Immunological assays.

HEK-Blue cells from InvivoGen stably express mNOD2 or hNOD2 along with an NF-κB–inducible secreted alkaline phosphatase. After stimulation, 5 μL cell culture supernatant was mixed with 45 μL QUANTI-Blue solution and incubated for 3 hours, followed by measurement of OD 630 nm. Mouse bone marrow macrophages (10^^5^^ cells/96-well plate) deficient in MyD88 or CARD2 were stimulated with LPS (10 ng/mL), cord factor (trehalose dimycolate [TDM]) (0.1 μg/well), or TbAd for 18–24 hours (38).

### Immunofluorescence microscopy.

BODIPY 493/503 (Thermo Fisher Scientific) was added for 1 hour to macrophages at 1 μg/mL. Coverslips were transferred onto a 24-well plate, washed in PBS, and fixed in 4% paraformaldehyde (PFA) (Electron Microscopy Services) for 20 minutes, washed in PBS, and incubated with anti-LAMP1 (BioLegend) or anti-LC3 (CST-4108S) diluted in permeabilizing buffer (10% FCS in PBS, 0.1% saponin) for 1 hour. Coverslips were washed and incubated with a secondary antibody for 30 minutes (1:1,000 dilution), followed by staining with 50 μg/mL Hoechst dye (MilliporeSigma) for 5–10 minutes. Coverslips were washed, mounted, and set overnight for imaging (Zeiss LSM 800 with Airyscan and Fiji). For the 1-TbAd dose response, cells were imaged directly without mounting.

AMs were incubated for 24 hours prior to treatment with TbAd (5–20 μM) for 2 hours and were then washed and cultured for 4–96 hours. Cells were fixed for 10 minutes with 4% PFA, washed with PBS, and permeabilized with 0.25% saponin for 1 hour at 20°C. Cells were incubated with blocking buffer (PBS 2% fish gelatin; MilliporeSigma) and 0.25 % saponin (MilliporeSigma) for 1 hour and incubated with mouse anti–human LAMP1 antibody (Santa Cruz Biotechnology, 1:100) overnight at 4°C. Cells were washed with 0.25% saponin in PBS and incubated with Alexa Fluor 594 goat anti–mouse IgG (Molecular Probes, 1:400 dilution) for 1 hour at room temperature and then washed. Alternatively, cells were stained with a 1:200 dilution of LipidTox Green (Invitrogen, Thermo Fisher Scientific) and Hoechst 33342 (10 μg/mL) in PBS for 30 minutes. Coverslips were mounted using DAKO antifade medium (Agilent Technologies) and imaged (Leica SP8 confocal microscope with CellProfiler 4.2.1).

RAW264.7 macrophages were seeded at 40,000 cells per well in 96-well Cellcarrier Ultra plates overnight and then washed in PBS and incubated in DMEM with 10% FCS containing 1:1,000 DMSO and 10 μM TbAd for 4 hours before fixing in 4% PFA, quenching (50 mM NH__4__Cl), permeabilizing (cold MeOH for 10 min), and blocking (PBS/0.5% BSA for 15 min). Primary antibodies in PBS/0.5% BSA were added for 1 hour at 20°C, followed by 3 washes in PBS and a secondary antibody incubation for 1 hour, and then 2 washes in PBS, incubation with DAPI (1:10,000), and washing in PBS. Cells were imaged (Opera Phenix) in confocal mode with 1 X binning. A *Z*-stack was captured from approximately 60 fields using Harmony (PerkinElmer). Cells were segmented using DAPI and Alex Fluor 488 for the Find Nuclei and Find Cytoplasm building blocks, respectively. LC3B and LAMP1 puncta were detected using the Find Spots building block. The following antibodies were used: LC3B (MBL International, PM036, 1:100), mouse LAMP1 (Developmental Studies, 1D4B, 1:100), human LAMP1 (Developmental Studies, H4A3, 1:100), anti–rabbit Alexa Fluor 488 (Thermo Fisher Scientific, A21441, 1:800), anti–rat Alexa Fluor 568 (Thermo Fisher Scientific, A11077, 1:800), and anti–mouse Alexa Fluor 568 (Thermo Fisher Scientific, A11004, 1:800).

### Autophagic flux.

RAW264.7 macrophages were electroporated with Neon (Life Technologies, Thermo Fisher Scientific) to express GFP-mCherry-LC3B (a gift from Sharon Tooze, The Francis Crick Institute, London, United Kingdom). Cells were washed in PBS and resuspended at 1.5 × 10^^6^^ cells in 100 μL Buffer R with 5 μg plasmid DNA before electroporating in Buffer E at 1,680 V for 20 ms and plated at 1 × 10^^5^^ cells per well in 96-well CellCarrier Ultra plates (PerkinElmer) overnight. Cells were washed twice in PBS and then stimulated with DMSO, 100 nM BafA1, and 10 μM TbAd for 4 hours before fixing in 4% PFA. Nuclei were counterstained with DAPI and more than 900 transfectants per condition were imaged (Opera Phenix).

### EM analysis.

Human macrophages (1 × 10^^6^^ cells/mL) in 6-well plates were fixed in 2% electron microscopy–grade PFA (MilliporeSigma) and 0.2% glutaraldehyde (GA) (MilliporeSigma) in 0.1 M PHEM buffer [240 mM piperazine-*N*,*N*′-bis(2-ethanesulfonic acid) in NaOH, pH 6.9, 100 mM HEPES, 8 mM MgCl__2__, 40 mM ethylene glycol tetraacetic acid]. Cells were washed in PBS with 0.02 M glycine and gently scraped using 1% gelatin in PBS and stored in PHEM with 0.5% PFA. After fixation and transport, cells were washed 3 times with PBS plus 0.02 M glycine (Merck) and embedded in 12% gelatin (MilliporeSigma) in 0.1 M phosphate buffer, followed by incubation for 5 minutes at 37˚C and vortexing every 2 minutes. After centrifugation for 3 minutes at 12,300*g* to pellet the cells in gelatin, the block was incubated for 10 minutes on ice. Blocks of approximately 1 × 1 mm were cut with a razor blade and incubated overnight in 2.3 M sucrose (Merck) in 0.1 M phosphate buffer prior to snap freezing. Samples were subjected to ultrathin (~60 nm) sectioning at –120°C or semithin sectioning (150–300 nm) at –100°C with a diamond knife (DiATOME, Cryo Immuno) on a Leica Ultracut UC6 cryoultramicrotome and transferred onto a formvar copper grid, gold finder grid, or glass slide in a droplet of 1:1 (*m/v*) 2% methylcellulose (MilliporeSigma) in 2.3 M sucrose and stored at 4°C.

### Transmission EM.

Grids were incubated on 2% gelatin in a 0.1 M phosphate buffer plate for 30 minutes at 37˚C and washed 5 times for 2 minutes. The grids were incubated with antibodies against LAMP1 (BD Pharmingen, H4A3, 1:50), perilipin-2 (PROGEN, 610102, 1:100), or LC3B (Abcam, ab48394, 1:10) in 1% BSA in PBS for 45 minutes. Then the grids were washed 5 times for 2 minutes with PBS containing 0.02 M glycine. For mouse antibodies, the grids were blocked for 3 minutes with 0.1% BSA in PBS with 0.02 M glycine and incubated for 20 minutes with rabbit anti-mouse antibody (DAKO, Z259, 1:200). For LAMP1 or perilipin-2 antibodies, cells were washed 6 times with PBS with 0.02 M glycine. The grids were blocked with 0.1% BSA in PBS with 0.02 M glycine and incubated for 20 minutes with protein A 10 nm gold (Utrecht University, 1:25) before washing 5 times with PBS, incubating for 5 minutes with 1% glutaraldehyde in PBS, and washing 10 times with Milli-Q water (MilliporeSigma). To contrast the samples, the grids were incubated with uranylacetate in 2% methylcellulose for 5 minutes and blotted. For double labeling, the sections were first immune-labeled with a monoclonal primary antibody and Protein-A 15 nm gold and subsequently with a polyclonal primary antibody and Protein-A 10 nm gold (Utrecht University, 1:50). The grids were imaged (Tecnai 120kV, Thermo Fisher Scientific) using a Velata and Xarosa (Emsis) camera with ImageJ Fiji (NIH).

### CLEM.

Using a modified method (77, 78), we washed cells 3 times for 5 minutes with PBS with 0.02 M glycine before incubating for 1 hour with anti-LAMP1 (Pharmingen, 1:50). We then washed cells 3 times for 10 minutes with PBS with 0.02 M glycine. The grids were incubated for 20 minutes with a goat anti–mouse Alexa Fluor 488 secondary antibody (Life technologies, Thermo Fisher Scientific, 1:500) and for 10 minutes with 1 mg/mL Nile red (MilliporeSigma, 1:25) and Hoechst 33342 (Thermo Fisher Scientific, 1:50). Finally, the grids were washed 5 times with PBS. The grids were mounted in between a glass slide and a coverslip in a droplet of VECTASHIELD and then imaged (Leica DM6, 100× oil objective). After wide-field imaging, the coverslip was removed, and the VECTASHIELD was washed from the grid with milliQ water at 37°C. Thereafter, the grids were contrasted using uranyl acetate in 2% methylcellulose for 5 minutes, blotted, and imaged (Fei Tecnai 120kV) with correlation (ICY Software) and CLEM (Huygens) deconvolution software.

### Immunofluorescence labeling of infected macrophages.

Semithin sections fixed for EM analysis on a glass slide were washed 3 times with PBS with 0.02 M glycine and incubated for 1 hour with anti–*M. tuberculosis* cell wall protein (C188, Colorado State University, 1:500) diluted in PBS with 0.1% BSA (MilliporeSigma), followed by washing 3 times for 10 minutes with PBS plus 0.02 M glycine, incubation for 20 minutes with goat anti–rabbit Alexa Fluor 488 (Molecular Probes), and, in the last 10 minutes, staining with Nile red (MilliporeSigma, 1:25) and Hoechst (Thermo Fisher Scientific, 1:50). Glass slides were washed 5 times with PBS before mounting with VECTASHIELD and imaged with a Leica DM6 microscope. To detect neutral lipids and phospholipids, semithin sections on a glass slide were washed 6 times with PBS with 0.02 M glycine and incubated with Nile red (MilliporeSigma, 1:25) and Hoechst 33342 (Thermo Fisher Scientific, 1:50) for 10 minutes. The slides were washed 5 times with PBS, mounted with VECTASHIELD, and imaged (Leica TCS SP8 confocal microscope).

### Enzyme bioactivity assays.

Human M1 macrophages were detached using 2 mM EDTA for 45 minutes and washed in PBS. Cells were pulsed with 240 μM C__12__FDG for 30 minutes and 200 μg/mL DQ-BSA (Thermo Fisher Scientific) for 15 minutes at 37°C, with Fixable Aqua (Invitrogen, Thermo Fisher Scientific) added in the final 10 minutes. Cells were washed before flow cytometry (BD Fortessa).

Human M1 macrophages were treated with TbAd (20 μM) and lalistat-2 (100 μM) in triplicate wells containing 3 × 10^^5^^ cells/well in a 24-well plate and then lysed using water containing 0.5% Triton X-100. Lysate (10 μL) was transferred onto a 96-well plate with 30 μL buffer containing 4-propyl-8-methyl-7-hydroxycoumarin (P-PMHC) (Cayman Chemical), an LAL substrate, incubated at 37°C for 3 hours, and quenched with 200 μL of 50% methanol. Lysate (150 μL) in a black flat-bottomed, 96-well plate was read (BioTek, Synergy H1 fluorimeter) at 360/460 nm excitation/emission wavelengths.

### Transcription of lysosomal hydrolases.

RNA extraction of M1 macrophages (RNeasy Plus kit, QIAGEN) were subjected to quantitative (QuantiNova Reverse Transcription Kit, QIAGEN) and real-time PCR (Agilent Technologies, AriaMx).

### Western blotting.

RAW264.7 macrophages (1 × 10^^5^^ cells/well in 12-well plates) were seeded overnight and washed twice with PBS, treated for 2–4 hours, washed in PBS, and then scraped into cold PBS. Starvation was performed in HBSS. BafA1 (100 nm), 1-TbAd (10 μM), or chloroquine at 10 μM treatment was followed by lysis (100 μL radioimmunoprecipitation assay buffer, MilliporeSigma) with protease and phosphatase inhibitor cocktail, Thermo Fisher Scientific) on ice for 10 minutes. Nuclei were pelleted at 13,000*g* for 5 minutes, and the supernatant was decanted. NuPAGE reducing agent and sample buffer (Thermo Fisher Scientific) were added, and the samples were boiled at 95°C for 10 minutes. The samples were run on 4%–12% SDS-PAGE gels and transferred onto PVDF membranes using iBlot2 and then blocked in 5% milk in TBS with 0.05% Tween-20 (TBS-T) for 1 hour at room temperature. Primary antibodies in 5% BSA in TBS-T were incubated overnight at 4°C, and then the membranes were washed 3 times in TBS-T. HRP-conjugated secondary antibodies were incubated for 1 hour at room temperature, washed 3 times before developing with enhanced chemiluminescent reagent (Merck) and imaging (Amersham AI600). The following antibodies were used: anti-LC3B (Abcam, 1:1,000), anti-p62 (Cell Signaling Technology, 1:1,000), anti–actin HRP (Cell Signaling Technology, 1:5,000), and anti–rabbit HRP (Promega, W4011, 1:10,000).

### HPLC-MS analysis of mammalian lipid extracts.

Macrophages were detached using Trypsin/EDTA (Gibco, Thermo Fisher Scientific) at 37°C for 30 minutes and then washed, and lipids were extracted in 2 mL 1:2 chloroform/methanol for 1 hour. Samples were pelleted, and supernatant was removed into separate glass vials. Then, the extraction was repeated with 2:1 chloroform/methanol. Supernatants were pooled and dried on glass under nitrogen. Lipids were resuspended in 2:1 chloroform/methanol and normalized to the cell number. A volume of 200 μL was dried under nitrogen and resuspended in 65 μL starting mobile phase (A). A volume of 10 μL was injected for reversed-phase positive mode HPLC-MS analysis (6530 Accurate-Mass Q-ToF, Agilent Technologies) using a Poroshell 120A, EC-C18 column (79), analyzed with MassHunter and XCMS software (47). To determine the structures of hexosylceramide, lipid samples were analyzed on an Inertsil Diol column (80). Collision energies of 20–40 V were used for CID-MS. HPLC-MS analysis of macrophage lipids was compared with known concentrations of lipid standards with the peak area plotted against concentration. For MADAGs, a TAG standard was used. Macrophage lipids were normalized to 4,000 cells/mL for injection of 10 μL.

### Reagents.

A list of the cell lines, chemicals, and antibodies used appears in Supplemental Table 1.

### Statistics.

Statistical analysis was performed using GraphPad Prism 8 (GraphPad Software), and R. Linear models were fitted using lme4 (81) and emmeans (https://CRAN.R-project.org/package=emmeans). One-way or factorial ANOVAs and 1-tailed *t* tests were used to determine statistical significance. A *P* value of less than 0.05 was used as the cutoff for significance.

### Study approval.

PBMCs (Mass General Brigham), AMs (St. James’ Hospital–Tallaght University Hospital), and mouse BMDMs (Harvard Medical Area Standing Committee on Animals) were obtained under institutional approval.

## Author contributions

MB, SVDN, EMB, GB, TYC, BA, AEG, SR, LB, EI, MPO, and SO conducted macrophage experiments. JAM performed the statistical analysis. JB and AJM produced synthetic lipids, and LOM and DSO provided lysosome activators. MB, JAM, AJM, SMF, DSO, JK, SY, MGG, NVDW, and DBM provided experimental oversight. MB, NVDW, MGG, and DBM conceived the project goals. The manuscript was written by MB, EMB, MGG, NVDW, and DBM, with input from all authors.

## Supplementary Material

Supplemental data

## Version 1 02/09/2023

In-Press Preview

## Version 2 03/15/2023

Electronic publication
