# Supplementary material for: A terpene nucleoside from M. tuberculosis induces lysosomal lipid storage in foamy macrophages
Source: J Clin Invest. 2023 Mar 15;133(6):e161944. doi: 10.1172/JCI161944 (PMC10014106; doi:10.1172/JCI161944)
Supplement: Supplemental data [file jci-133-161944-s165.pdf]

List of Supplemental Figures and Table

Supplemental Figure 1. Synthetic 1-TbAd does not detectably activate immune response.

Supplemental Figure 2. 1-TbAd induces dose-dependent swelling of human macrophage lysosomes.

Supplemental Figure 3. 1-TbAd induces selective swelling of lysosomes in human macrophages.

Supplemental Figure 4. Fluorescence microscopy analysis of human alveolar macrophages (AM).

Supplemental Figure 5. Western blotting of autophagy markers in response to lysosomally active compounds.

Supplemental Figure 6. Summary of 1-TbAd induced lipid change in human macrophages.

Supplemental Figure 7. Quantitation of 1-TbAd induced neutral lipids.

Supplemental Figure 8. 1-TbAd induced lipid changes in membrane lipids.

Supplemental Figure 9. 1-TbAd influence on Ganglioside M3 (GM3) and lactosyl ceramide.

Supplemental Figure 10. HPLC analysis of an unknown macrophage-derived lipid.

Supplemental Figure 11. Analysis of lysosomal swelling in response to Mtb infection over time.

Supplemental Table 1. Listing of sources of antibodies, chemicals and cell lines.

References for Supplemental Figures

Buter, J., Cheng, T.Y., Ghanem, M., Grootemaat, A.E., Raman, S., Feng, X., Plantijn, A.R., Ennis, T., Wang, J., Cotton, R.N., *et al.* (2019). Mycobacterium tuberculosis releases an antacid that remodels phagosomes. Nat Chem Biol *15*, 889-899.

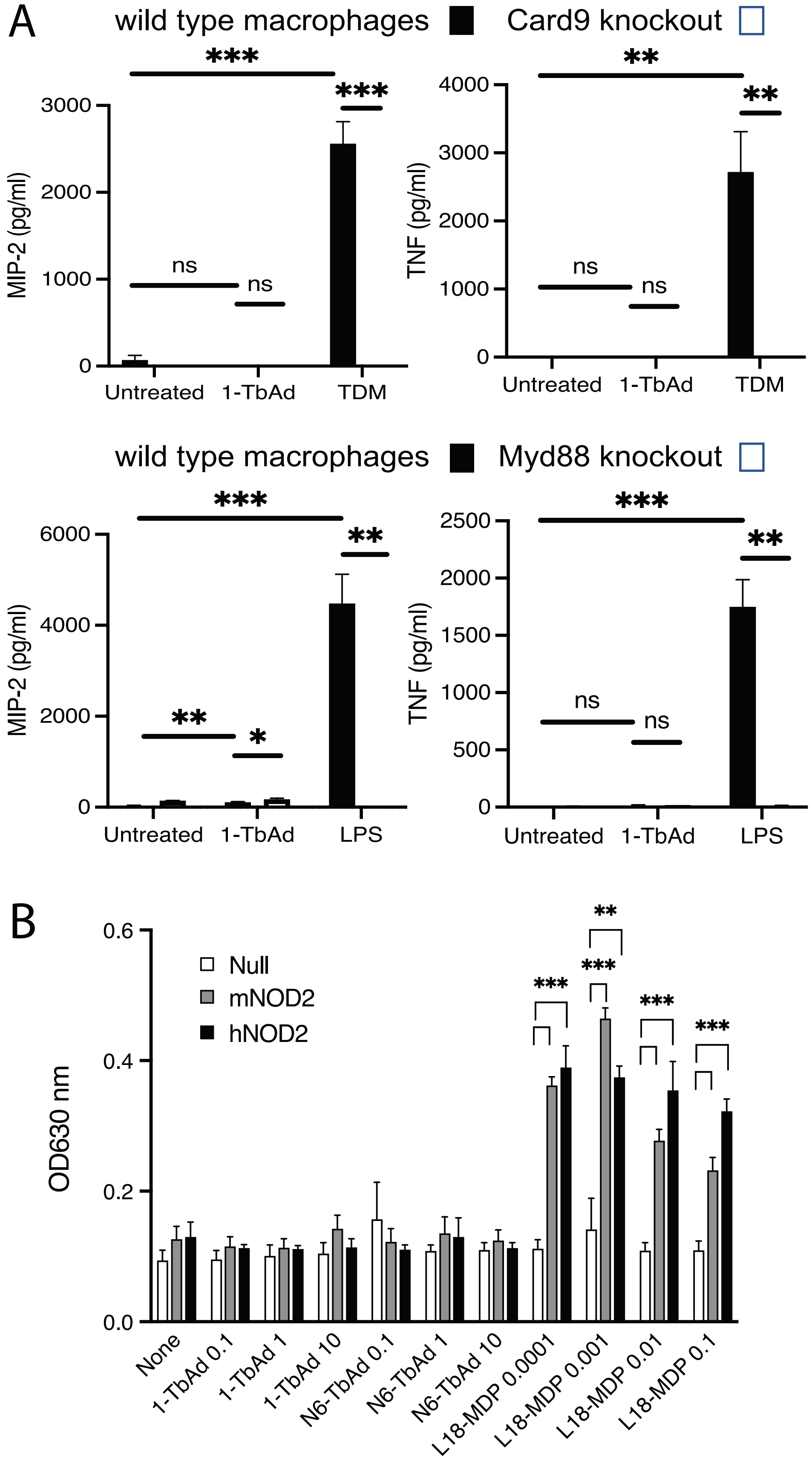

Supplemental Figure 1. Synthetic 1-TbAd does not detectably activate immune response. A. Wild-type, CARD9-and MyD88- macrophages were treated with 100 ng/well 1-TbAd, 10 ng/ml lipopolysaccharide (LPS), or 100 ng/well trehalose dimycolate (TDM) for 24 hours and subjected to ELISA. B. HEK-Blue cells (Invivogen) stably expressing mNOD2 or hNOD2 along with an NF-κB-inducible secreted alkaline phosphatase were stimulated with muramyl dipeptide (L18-MDP) or TbAd with the indicated micromolar dose. After stimulation, 5 µl of cell culture supernatant was subject to a colorimetric enzymatic activity assay. Assays are representative of two or more experiments analyzed with the unpaired two-tailed Student's t-test (*, $p<0.05$; **, $p<0.01$;***, $p<0.001$).

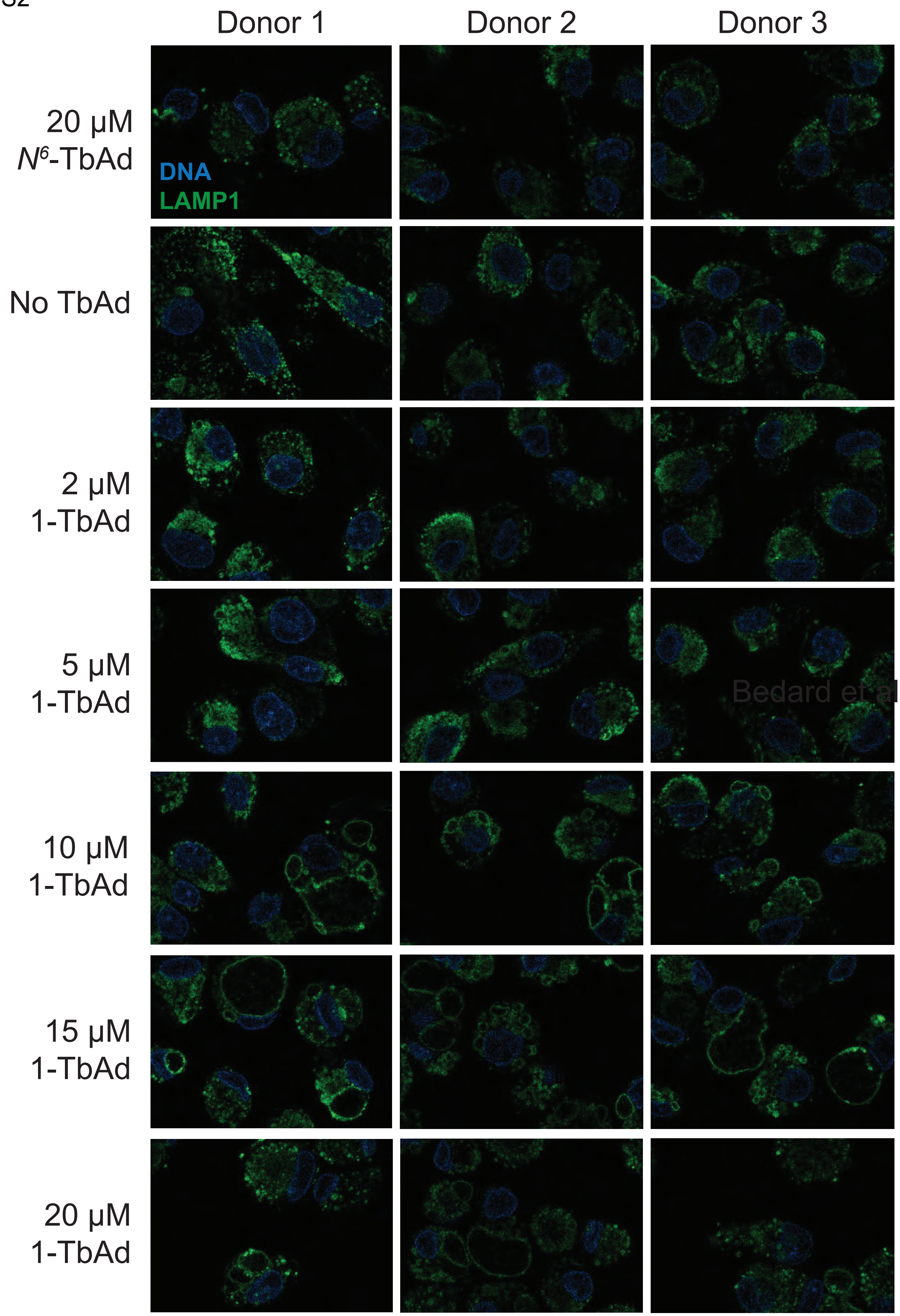

Bedard et al.

Supplemental Figure 2. 1-TbAd induces dose-dependent swelling of human macrophage lysosomes. Representative images of LAMP1 staining following 4 hours of treatment with varying TbAd concentrations in human M1 macrophages from 3 human donors. LAMP1 compartments in untreated cells appear as puncta because they lack visible lumens, but 1-TbAd treated cells show large (0.5-5 μm) LAMP1 rings indicative of lysosomal swelling.

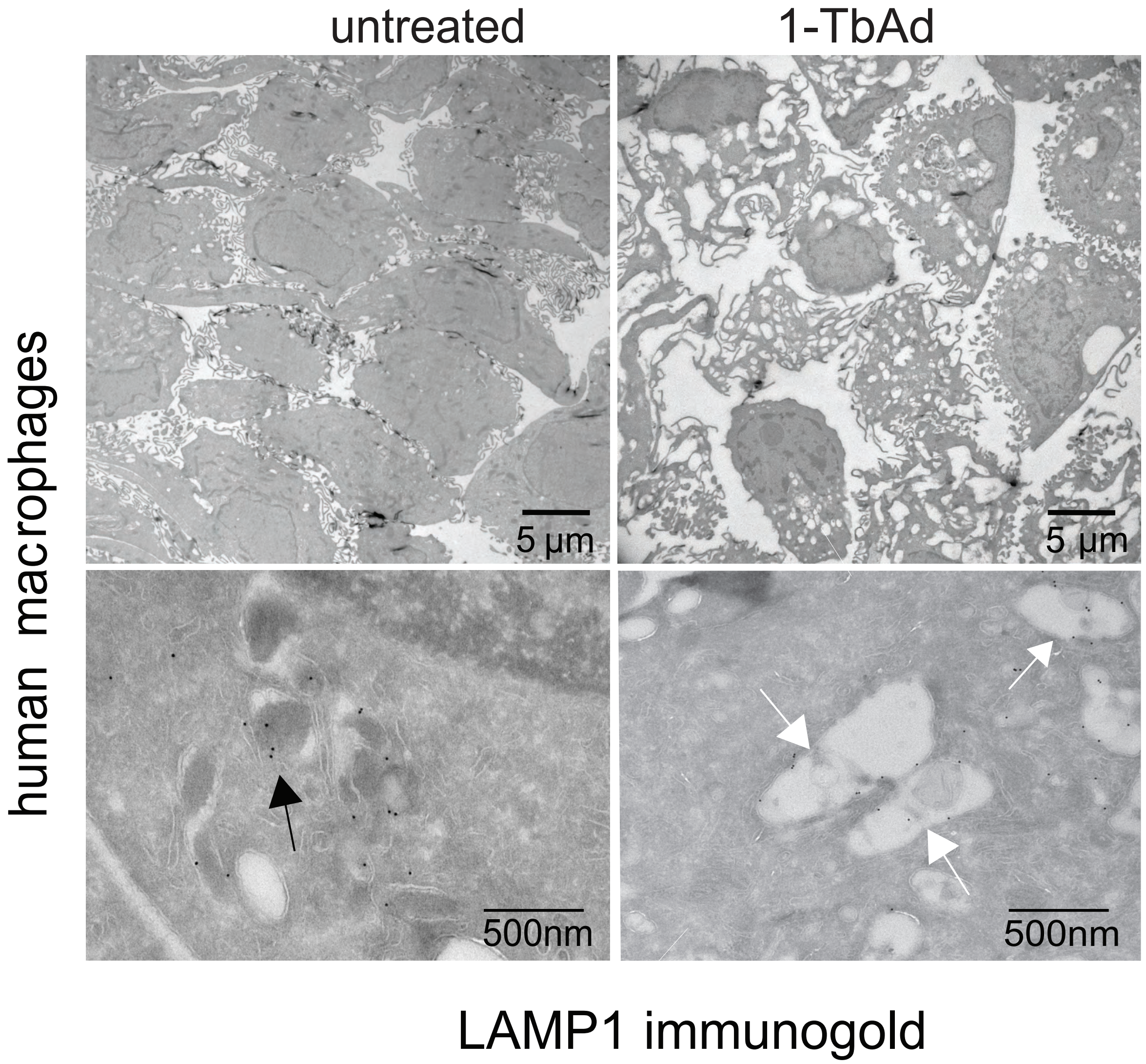

Supplemental Figure 3. 1-TbAd induces selective swelling of lysosomes in human macrophages. Low (upper panels) and high (lower panels) magnification of images of human M1 macrophages showed many small electron dense compartments, whereas cells treated with 10µM 1-TbAd for 4 hours show loss of electron dense compact compartments and the appearance of large electron-lucent compartments. As contrasted to compact (~200 nm) electron dense lysosomes (black arrows), swollen (>500 nm) LAMP-1+ (black immunogold) lysosomes were electron lucent with intralumenal particulates (white arrows).

Figure S4

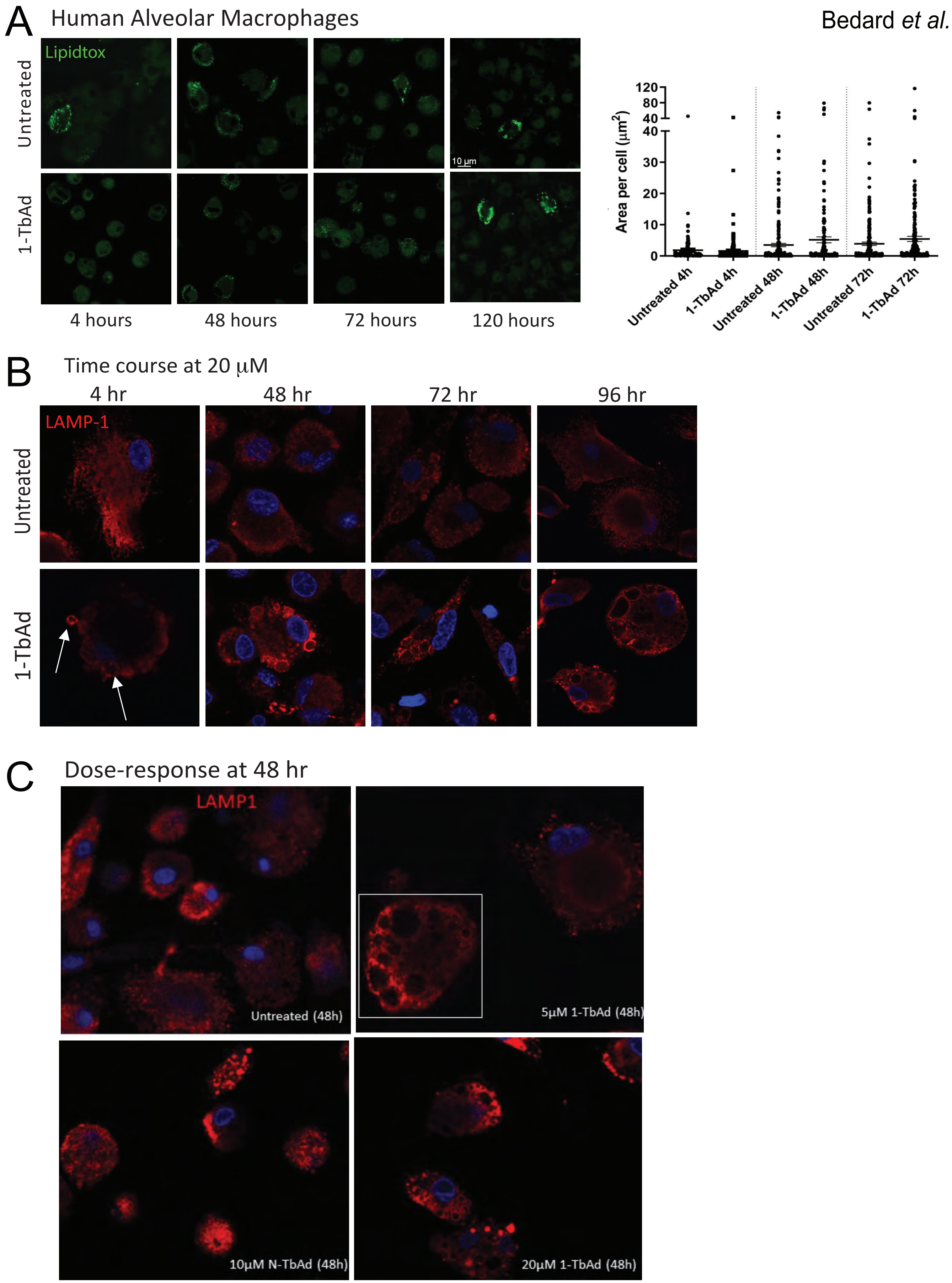

Supplemental Figure 4. Fluorescence microscopy analysis of human alveolar macrophages (AM).
A. Representative images and quantification from LipidTox green staining of fresh AM from bronchoscopy of four human subjects; cells treated with 1-TbAd (20µM) or untreated for 2 hours, then chased, fixed, and treated with LipidTox for the indicated time. B-C. Human alveolar macrophages were treated with 1-TbAd at the indicated dose and time and subjected to anti-LAMP1 staining and immunofluorescence microscopy; whereas lysosomes from untreated cells appear as puncta, large (0.5-5 µm) rings represent swollen lysosomal compartments. The 48 hour time point in the B panel is also shown in Figure 1F.

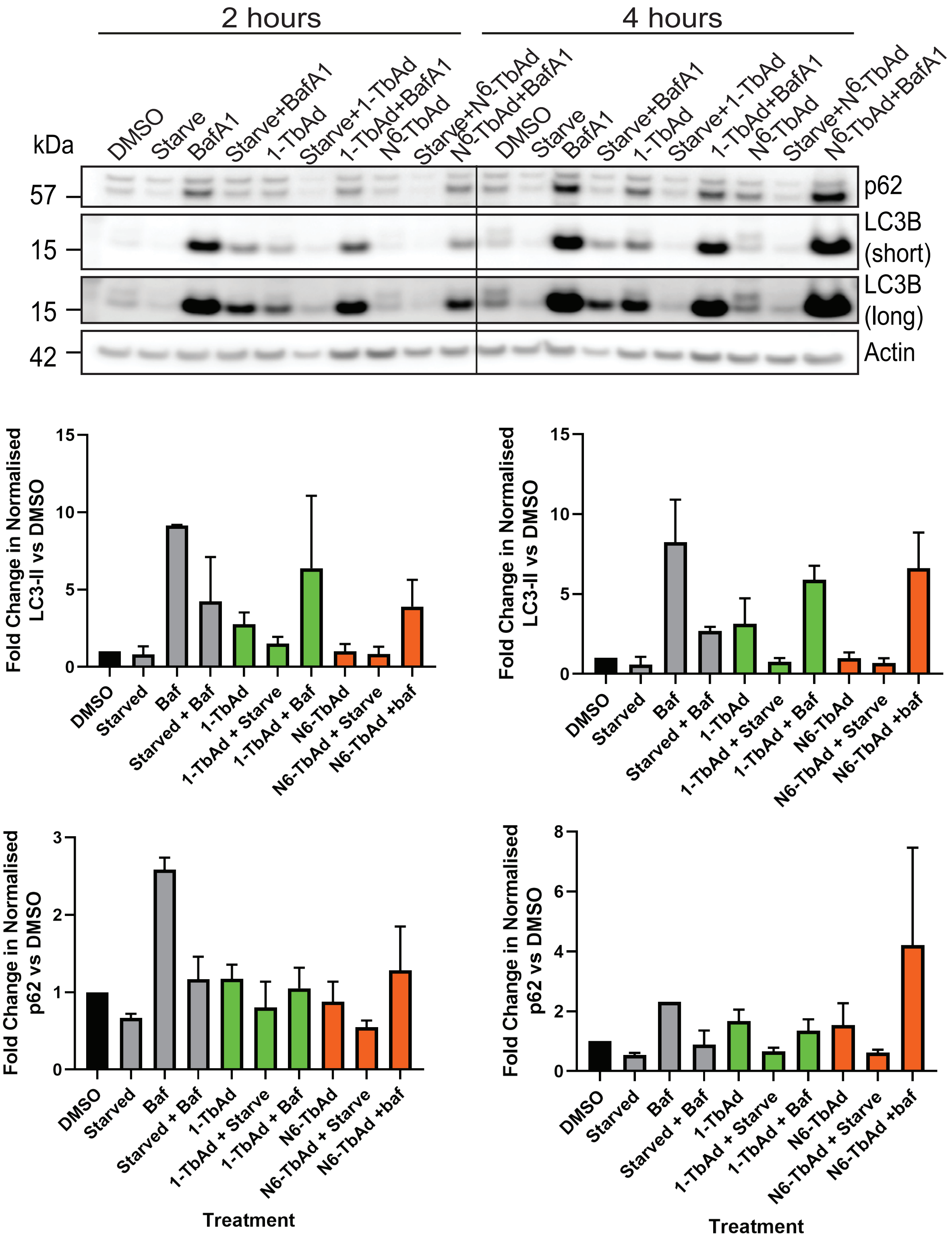

Supplemental Figure 5. Western blotting of autophagy markers in response to lysosomally active compounds. Mouse macrophages were treated with nutrient-free Hank's buffered saline (starvation), chloroquine (CQ), dimethyl sulfoxide vehicle (DMSO), 1-TbAd, *N*[6]-TbAd, or Baf A1 for 2 or 4 hours, followed by blotting for p62, LC3-II, or actin loading control, in three separate experiments; quantification of bands relative to vehicle (DMSO) control shown for experiment 3.

Unidentified total: 13 species

	detected *m/z*	RT (min)		detected *m/z*	RT (min)
1	502.2934	3.066	8	580.3818	3.865
2	578.3097	3.227	9	496.3385	4.112
3	584.3607	3.272	10	426.3548	4.635
4	580.3648	3.301	11	860.5415	5.897
5	566.3497	3.301	12	318.3138	10.486
6	275.2567	3.707	13	840.5735	11.291
7	332.3290	3.831			

Known 6 lipid classes total: 92 species

cholesteryl ester (CE)

	detected *m/z*	RT (min)	formula	calculated *m/z*	lipid chain
1	690.6170	14.796	C47H80NO2 [M+NH4]+	690.6184	20:4
2	666.6176	14.886	C45H80NO2 [M+NH4]+	666.6184	18:2
3	692.6318	14.907	C47H82NO2 [M+NH4]+	692.6340	20:3
4	668.6330	15.070	C45H82NO2 [M+NH4]+	668.6340	18:1
5	642.6189	15.099	C43H80NO2 [M+NH4]+	642.6184	16:0

hexsosylceramide (HexCer)

	detected *m/z*	RT (min)	formula	calculated *m/z*	lipid chain
1	700.5694	11.601	C40H78NO8 [M+H]+	700.5722	34:1

phosphatidylinositol (PI)

	detected *m/z*	RT (min)	formula	calculated *m/z*	lipid chain
1	882.6040	11.246	C45H89NO13P [M+NH4]+	882.6066	36:1

tuberculosinyladenosine (TbAd)

	detected *m/z*	RT (min)	formula	calculated *m/z*
1-TbAd	540.3538	3.272	C30H46N5O4 [M+H]+	540.3544
N6-TbAd	540.3532	5.688	C30H46N5O4 [M+H]+	540.3545

triacylglycerol (TAG)

	detected *m/z*	RT (min)	formula	calculated *m/z*	lipid chain
1	968.7694	14.088	C63H102NO6 [M+NH4]+	968.7702	C60:12
2	994.7844	14.088	C65H104NO6 [M+NH4]+	994.7858	C62:13
3	944.7688	14.160	C61H102NO6 [M+NH4]+	944.7702	C58:10
4	970.7836	14.170	C63H104NO6 [M+NH4]+	970.7859	C60:11
5	1022.8180	14.185	C67H108NO6 [M+NH4]+	1022.8171	C64:13
6	996.7998	14.196	C65H106NO6 [M+NH4]+	996.8015	C62:12
7	920.7695	14.226	C59H102NO6 [M+NH4]+	920.7702	C56:8
8	946.7824	14.246	C61H104NO6 [M+NH4]+	946.7858	C58:9
9	972.7975	14.283	C63H106NO6 [M+NH4]+	972.8015	C60:10
10	1024.8310	14.301	C67H110NO6 [M+NH4]+	1024.8328	C64:12
11	998.8142	14.303	C65H108NO6 [M+NH4]+	998.8171	C62:11
12	896.7684	14.333	C57H102NO6 [M+NH4]+	896.7702	C54:6
13	922.7844	14.364	C59H104NO6 [M+NH4]+	922.7858	C56:7
14	884.7695	14.374	C56H102NO6 [M+NH4]+	884.7702	C53:5
15	948.7996	14.377	C61H106NO6 [M+NH4]+	948.8015	C58:8
16	974.8139	14.388	C63H108NO6 [M+NH4]+	974.8187	C60:9
17	910.7857	14.398	C58H104NO6 [M+NH4]+	910.7858	C55:6
18	872.7681	14.402	C55H102NO6 [M+NH4]+	872.7702	C52:4
19	936.8012	14.413	C60H106NO6 [M+NH4]+	936.8015	C57:7
20	1000.8250	14.414	C65H110NO6 [M+NH4]+	1000.8328	C62:10
21	898.7834	14.442	C57H104NO6 [M+NH4]+	898.7858	C54:5
22	886.7828	14.468	C56H104NO6 [M+NH4]+	886.7858	C53:4
23	924.8001	14.468	C59H106NO6 [M+NH4]+	924.8015	C56:6
24	950.8141	14.485	C61H108NO6 [M+NH4]+	950.8171	C58:7
25	976.8296	14.496	C63H110NO6 [M+NH4]+	976.8328	C60:8
26	848.7685	14.505	C53H102NO6 [M+NH4]+	848.7702	C50:2
27	874.7842	14.511	C55H104NO6 [M+NH4]+	874.7858	C52:3
28	912.7990	14.514	C58H106NO6 [M+NH4]+	912.8015	C55:5
29	938.8153	14.516	C60H108NO6 [M+NH4]+	938.8171	C57:6
30	1002.8440	14.535	C65H112NO6 [M+NH4]+	1002.8484	C62:9
31	900.7987	14.545	C57H106NO6 [M+NH4]+	900.8015	C54:4
32	862.7841	14.582	C54H104NO6 [M+NH4]+	862.7858	C51:2
33	926.8144	14.583	C59H108NO6 [M+NH4]+	926.8171	C56:5
34	888.7990	14.588	C56H106NO6 [M+NH4]+	888.8015	C53:3
35	952.8292	14.597	C61H110NO6 [M+NH4]+	952.8328	C58:6
36	914.8136	14.622	C58H108NO6 [M+NH4]+	914.8171	C55:4
37	978.8465	14.630	C63H112NO6 [M+NH4]+	978.8484	C60:7
38	940.8305	14.637	C60H110NO6 [M+NH4]+	940.8328	C57:5
39	1005.8610	14.656	C65H114NO6 [M+NH4]+	1004.8641	C62:8
40	902.8153	14.673	C57H108NO6 [M+NH4]+	902.8171	C54:3
41	876.8007	14.681	C55H106NO6 [M+NH4]+	876.8015	C52:2
42	928.8288	14.705	C59H110NO6 [M+NH4]+	928.8328	C56:4
43	954.8439	14.730	C61H112NO6 [M+NH4]+	954.8484	C58:5
44	942.8453	14.731	C60H112NO6 [M+NH4]+	942.8484	C57:4
45	980.8594	14.734	C63H114NO6 [M+NH4]+	980.8641	C60:6
46	916.8294	14.741	C58H110NO6 [M+NH4]+	916.8328	C55:3
47	864.7987	14.743	C54H106NO6 [M+NH4]+	864.8015	C51:1
48	890.8150	14.744	C56H108NO6 [M+NH4]+	890.8171	C53:2
49	930.8462	14.828	C59H112NO6 [M+NH4]+	930.8484	C56:3
50	904.8308	14.838	C57H110NO6 [M+NH4]+	904.8328	C54:2
51	956.8584	14.848	C61H114NO6 [M+NH4]+	956.8641	C58:4
52	892.8296	14.857	C56H110NO6 [M+NH4]+	892.8328	C53:1
53	878.8156	14.865	C55H108NO6 [M+NH4]+	878.8171	C52:1
54	982.8753	14.899	C63H116NO6 [M+NH4]+	982.8797	C60:5
55	1008.8910	14.906	C65H118NO6 [M+NH4]+	1008.8954	C62:6
56	984.8913	14.997	C63H118NO6 [M+NH4]+	984.8954	C60:4
57	932.8613	15.006	C59H114NO6 [M+NH4]+	932.8641	C56:2
58	986.9079	15.148	C63H120NO6 [M+NH4]+	986.9110	C60:3
59	1038.9400	15.192	C67H124NO6 [M+NH4]+	1038.9423	C64:5
60	988.9242	15.318	C63H122NO6 [M+NH4]+	988.9267	C60:2
61	962.9088	15.331	C61H120NO6 [M+NH4]+	962.9110	C58:1

monoalkyldiacylglycerol (MADAG)

	detected *m/z*	RT (min)	formula	calculated *m/z*	lipid chain
1	908.8064	14.658	C59H106NO5 [M+NH4]+	908.8066	C56:7
2	882.7861	14.661	C57H104NO5 [M+NH4]+	882.7909	C54:6
3	858.7869	14.741	C55H104NO5 [M+NH4]+	858.7909	C52:4
4	884.8020	14.761	C57H106NO5 [M+NH4]+	884.8066	C54:5
5	910.8193	14.767	C59H108NO5 [M+NH4]+	910.8222	C56:6
6	936.8406	14.777	C61H110NO5 [M+NH4]+	936.8379	C58:7
7	834.7879	14.814	C53H104NO5 [M+NH4]+	834.7909	C50:2
8	860.8030	14.826	C55H106NO5 [M+NH4]+	860.8066	C52:3
9	886.8175	14.859	C57H108NO5 [M+NH4]+	886.8222	C54:4
10	912.8334	14.889	C59H110NO5 [M+NH4]+	912.8379	C56:5
11	938.8520	14.923	C61H112NO5 [M+NH4]+	938.8535	C58:6
12	862.8200	14.959	C55H108NO5 [M+NH4]+	862.8222	C52:2
13	836.8042	14.973	C53H106NO5 [M+NH4]+	836.8066	C50:1
14	888.8344	14.977	C57H110NO5 [M+NH4]+	888.8379	C54:3
15	914.8461	14.984	C59H112NO5 [M+NH4]+	914.8535	C56:4
16	940.8646	15.034	C61H114NO5 [M+NH4]+	940.8692	C58:5
17	966.8828	15.064	C63H116NO5 [M+NH4]+	966.8848	C60:6
18	916.8640	15.119	C59H114NO5 [M+NH4]+	916.8692	C56:3
19	890.8497	15.122	C57H112NO5 [M+NH4]+	890.8535	C54:2
20	942.8793	15.150	C61H116NO5 [M+NH4]+	942.8848	C58:4
21	968.8947	15.193	C63H118NO5 [M+NH4]+	968.9005	C60:5
22	970.9098	15.309	C63H120NO5 [M+NH4]+	970.9161	C60:4

Supplemental Figure 6. Summary of 1-TbAd induced lipid change in human macrophages. Lipids are described according to their chemical names, or—for detected molecules of unknown composition—by their m/z values. Raw intensity values are given for every lipid, and selected lipids of high biological value were compared to external standard curves to generate absolute lipid concentration. Lipids are considered changed by 1-TbAd treatment when their intensity value changes by more than 2-fold with a multiple-test-corrected p-value < 0.05.

external standard

cholesteryl ester (CE)

$Y = -73887X^2 + 485516X + 5176$

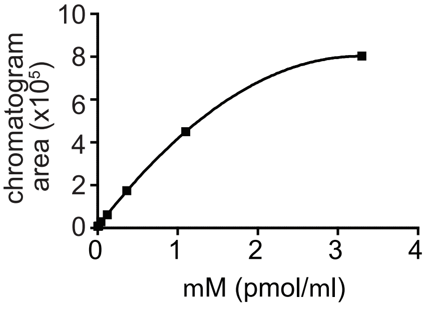

triacylglycerol (TAG)

$Y = 4845000X + 1334$

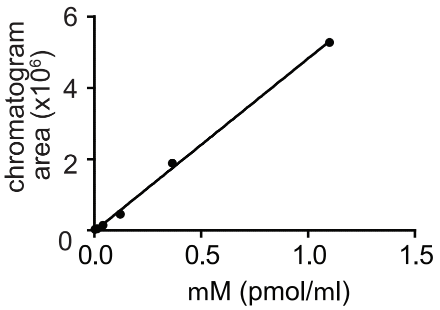

macrophage lipids

intensity (counts)

m/z (lipid chain)	untreated	N6-TbAd	1-TbAd 5μM	1-TbAd 10μM	1-TbAd 20μM
642.6189 (16:0)	60422±8136	57248±5097	109938±21760	114756±39665	118544±7720
666.6176 (18:2)	68054±10147	6220±6618	121474±33040	137781±48426	141095±6882
668.6330 (18:1)	155807±18909	142712±34887	347662±85469	444755±126677	417446±66729
690.6184 (20:4)	74903±8612	63134±13551	163638±33473	255550±139198	247460±58703
**pmol / million cells**					
642.6189 (16:0)	289.7±43.5	272.7±27.2	559.1±120.3	587.2±221.3	606.2±42.9
666.6176 (18:2)	330.5±54.6	299.1±35.4	623.9±184.3	717.2±275.5	732.6±39.0
668.6330 (18:1)	816.7±107.7	743.4±198.8	2026.7±569.2	2765.3±946.4	2518.5±485.7
690.6184 (20:4)	367.3±46.4	304.3±72.6	862.7±191.4	1450.5±904.1	1366.5±359.0

intensity (counts)

m/z (lipid chain)	untreated	N6-TbAd	1-TbAd 5μM	1-TbAd 10μM	1-TbAd 20μM
848.7702 (50:2)	168176±1454	246398±34547	229499±161063	355008±129199	394242±54882
876.8015 (52:2)	450822±29725	559546±94582	970576±137714	991938±415657	999044±74712
898.7858 (54:5)	96757±10938	107807±31586	431051±265473	380241±98427	541210±102987
928.8328 (56:4)	109463±21518	126164±22942	349792±184535	250693±77012	401152±31257
**pmol / million cells**					
848.7702 (50:2)	100.3±0.8	140.6±17.8	131.9±83.1	196.7±66.7	216.9±28.3
876.8015 (52:2)	246.1±15.3	302.2±48.8	514.3±71.1	525.3±214.5	529.0±38.6
898.7858 (54:5)	63.4±5.6	69.1±16.3	235.9±137.0	209.7±50.8	292.7±53.1
928.8328 (56:4)	70.0±11.1	78.6±11.8	194.0±95.2	142.8±39.7	220.5±16.1

The estimated lipid pool size by HPLC-MS analysis was obtained by comparing MS counts of macrophage lipids to the indicated external standards, as detailed in the Methods.

Supplemental Figure 7. Quantitation of 1-TbAd induced neutral lipids. To estimate the neutral lipid pool sizes based on lipid concentration, authentic lipids were obtained and used to generate standard curves.

external standard

macrophage lipids

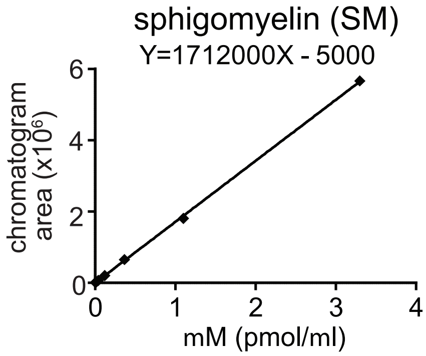

intensity (counts)

m/z (lipid chain)	untreated	N6-TbAd	1-TbAd 5µM	1-TbAd 10µM	1-TbAd 20µM
703.5749 (34:1)	3209955±47299	3581229±623502	4560278±408156	4358750±629302	3298152±365416
**pmol / million cells**					
703.5749 (34:1)	4694.7±69.1	5236.9±910.5	6666.6±596.0	6372.3±919.0	4823.5±533.6

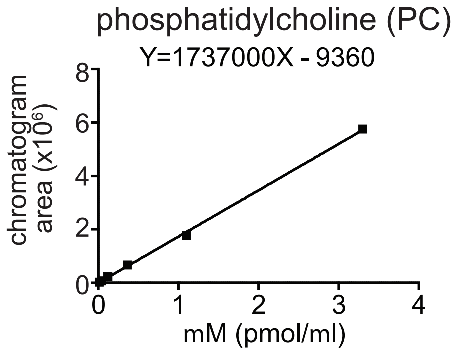

intensity (counts)

m/z (lipid chain)	untreated	N6-TbAd	1-TbAd 5µM	1-TbAd 10µM	1-TbAd 20µM
760.5851 (34:1)	4985632±187364	5487286±691158	5930169±549375	5066947±331477	4345116±127015
**pmol / million cells**					
760.5851 (34:1)	7189.1±269.7	7911.1±994.8	8548.5±790.7	7306.1±477.1	6267.2±182.8

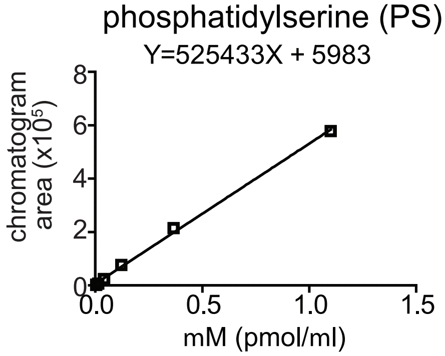

intensity (counts)

m/z (lipid chain)	untreated	N6-TbAd	1-TbAd 5µM	1-TbAd 10µM	1-TbAd 20µM
762.5280 (34:1)	95497±8173	122292±22968	126854±16018	117193±20543	96167±2014
**pmol / million cells**					
762.5280 (34:1)	425.9±38.9	553.4±109.3	575.1±76.2	529.1±97.7	429.1±9.6

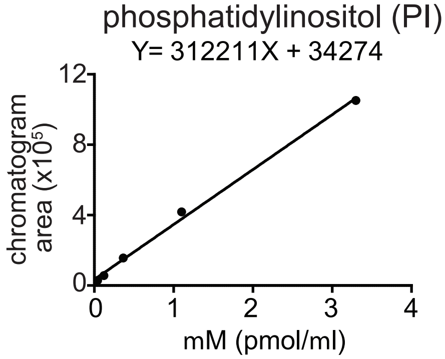

intensity (counts)

m/z (lipid chain)	untreated	N6-TbAd	1-TbAd 5µM	1-TbAd 10µM	1-TbAd 20µM
882.6067 (36:1)	53346±2267	67133±13328	75505±6449	90712±5144	132560±8877
**pmol / million cells**					
882.6067 (36:1)	261.4±15.4	355.0±90.5	411.9±43.8	515.1±34.9	799.3±60.3

Supplemental Figure 8. 1-TbAd induced lipid changes in membrane lipids. To estimate the membrane phospholipid pool sizes based on lipid concentration, authentic standards for selected lipids were obtained and used to generate standard curves.

Figure S9

Bedard *et al.*

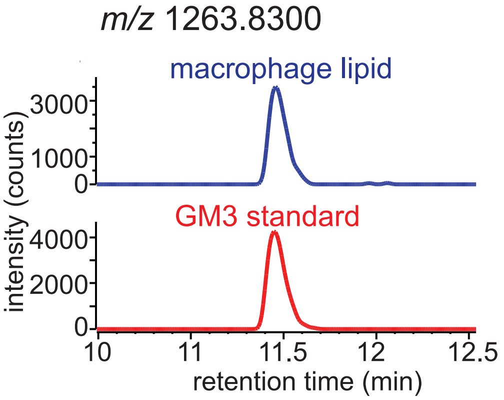

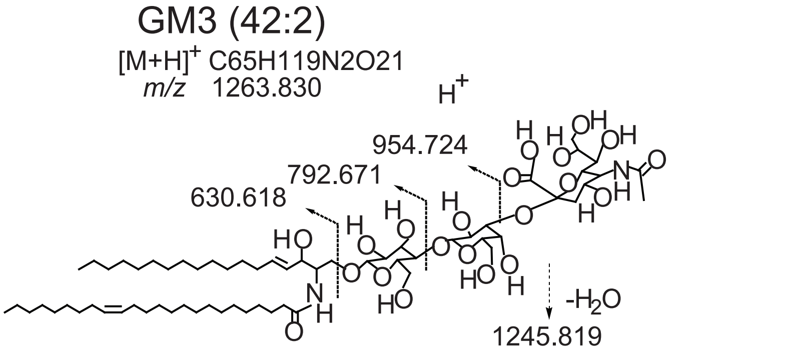

external standard

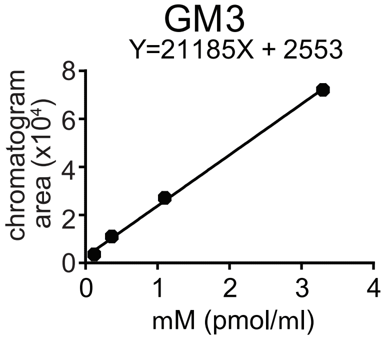

macrophage lipids

GM3 intensity (counts)

m/z (lipid chain)	untreated	N6-TbAd	1-TbAd 5µM	1-TbAd 10µM	1-TbAd 20µM
1263.8300(42:2)	21848±1610	28940±5736	32098±2294	30319±4229	23902±317
**pmol / million cells**					
1263.8300(42:2)	2277±190	3114±677	3487±271	3277±499	2519±37

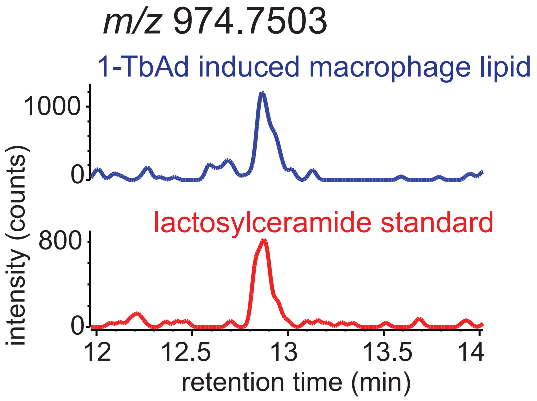

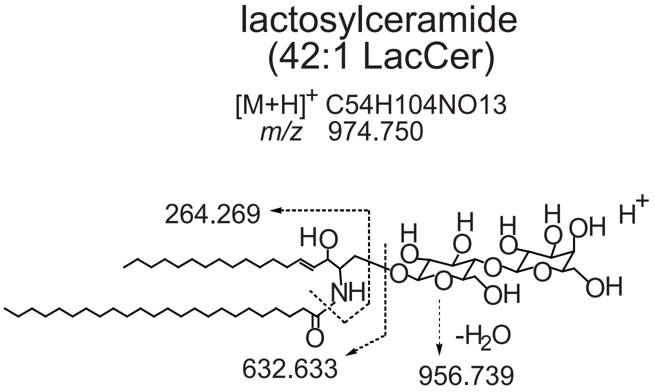

external standard

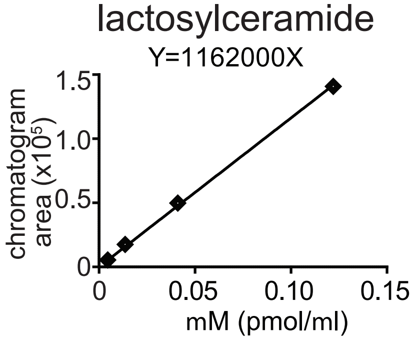

macrophage lipids

dihexsosylceramide intensity (counts)

m/z (lipid chain)	untreated	N6-TbAd	1-TbAd 5µM	1-TbAd 10µM	1-TbAd 20µM
974.7503 (42:1)	1644±393	1863±878	2246±843	2084±615	9182±1610
972.7351 (42:2)	2063±515	1677±756	2482±567	3780±469	13218±1356
**pmol / million cells**					
974.7503 (42:1)	3.5±0.8	4.0±1.9	4.8±1.8	4.5±1.3	19.8±3.5
972.7351 (42:2)	4.4±1.1	3.6±1.6	5.3±1.2	8.1±1.0	28.4±2.9

Supplemental Figure 9. 1-TbAd influence on Ganglioside M3 (GM3) and lactosyl ceramide. HPLC analysis of human macrophage derived lipids with authentic standards shows coelution. CID-MS of both macrophage-derived lipids in the positive mode shows dehydradation products or loss of the indicated carbohydrate or lipid moiety. External standard curves were generated and used to estimate the absolute concentration of lipid induced.

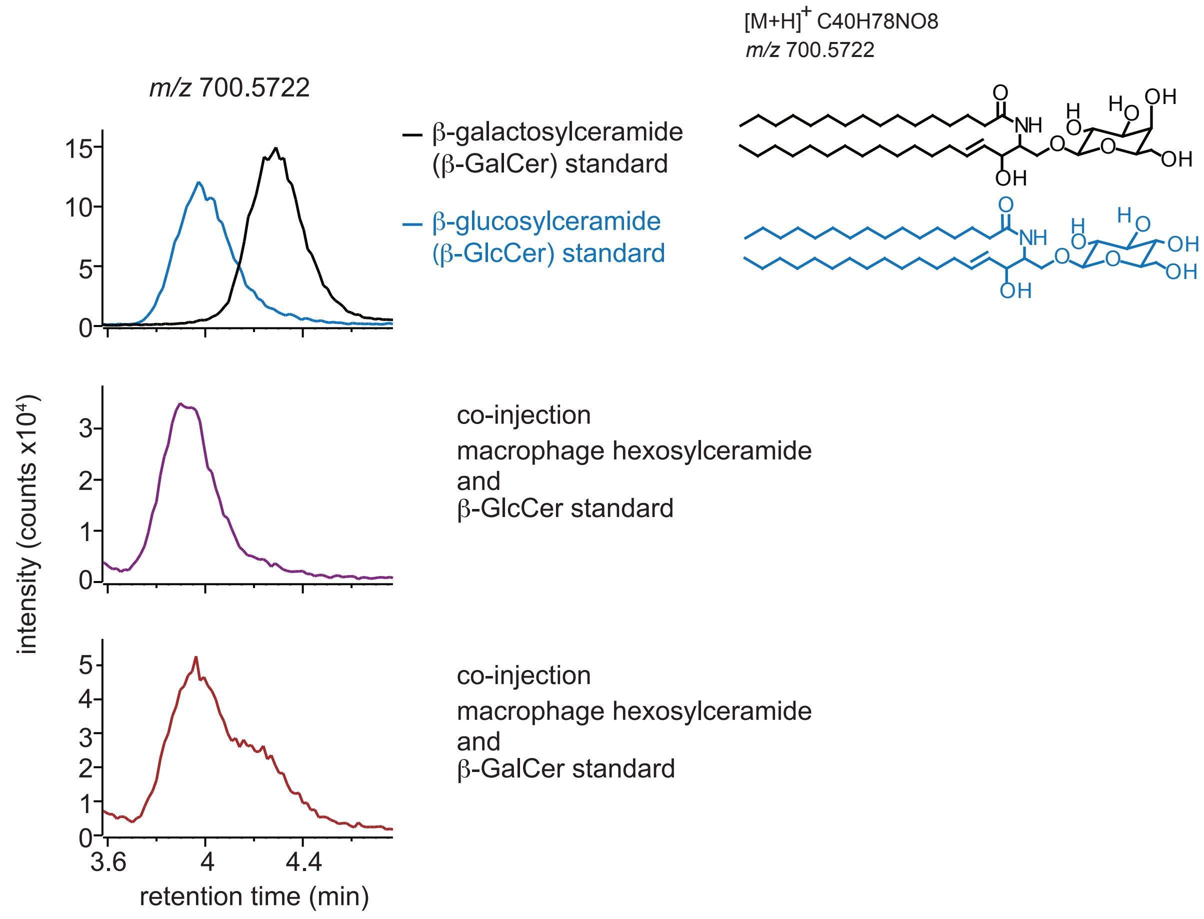

external standard

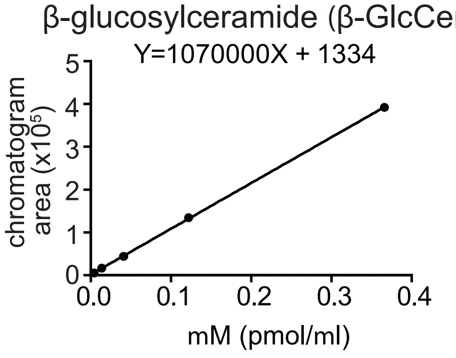

hexsosylceramide intensity (counts)

m/z (lipid chain)	untreated	N6-TbAd	1-TbAd 5μM	1-TbAd 10μM	1-TbAd 20μM
700.5722 (34:1)	7695±1413	9929±3181	13180±2539	15230±3397	27110±3644
**pmol / million cells**					
700.5722 (34:1)	14.9±3.3	20.1±7.4	27.7±5.9	32.5±7.9	60.2±8.5

Supplemental Figure 10. HPLC analysis of an unknown macrophage-derived lipid. Comparison to an internal β-glucosyl ceramide shows precise comigration of the standard and unknown. External standard curves were generated and used to estimate the absolute lipid concentration.

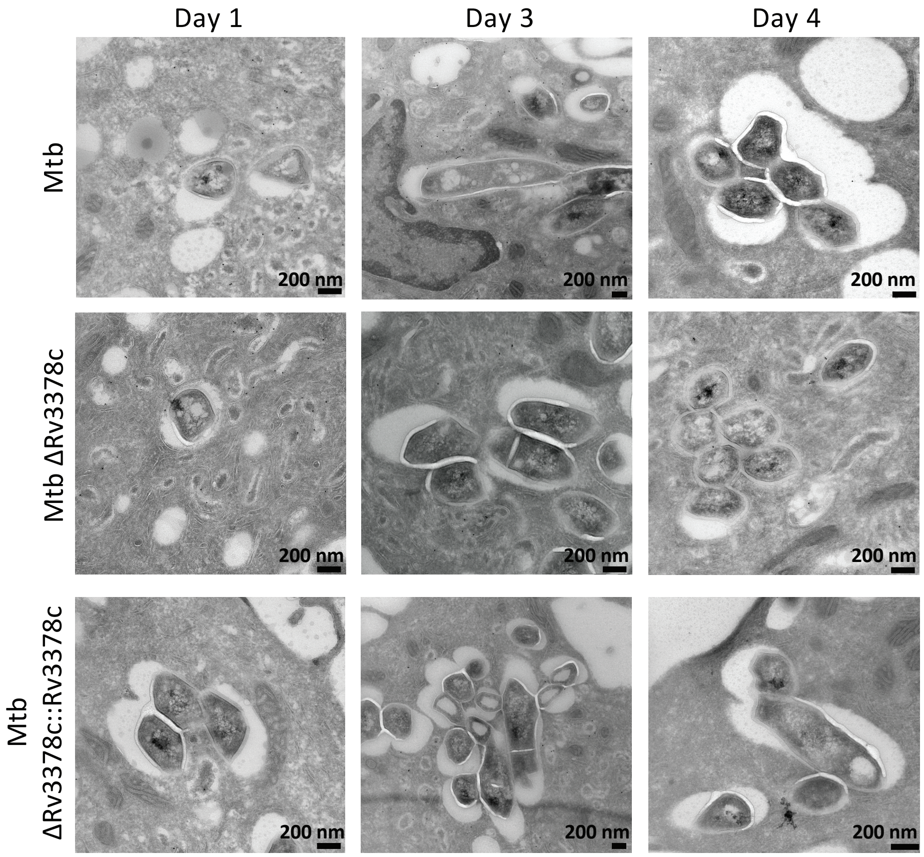

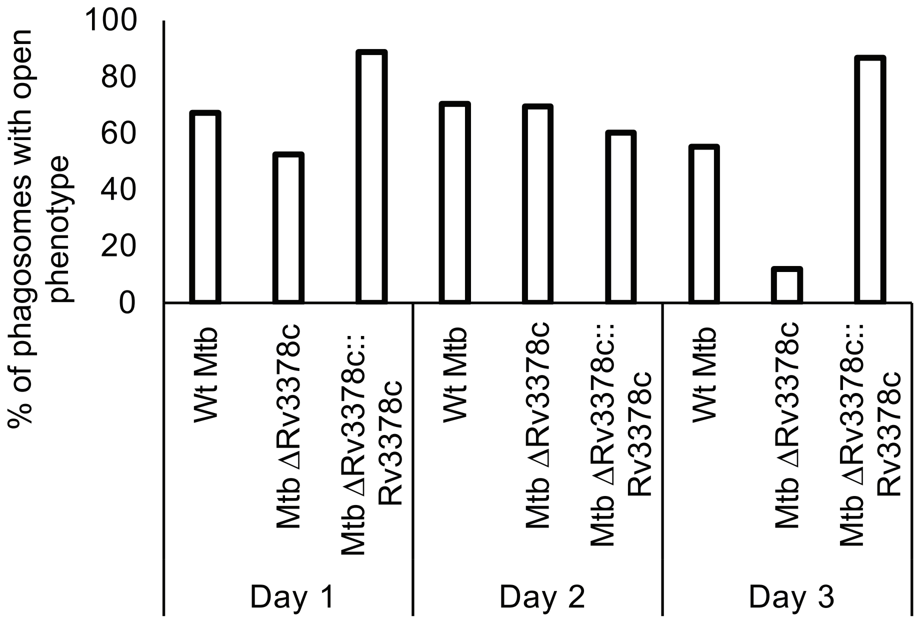

Supplemental Figure 11. Analysis of lysosomal swelling in response to Mtb infection over time. Human macrophages were infected with the indicated strain of Mtb H37Rv (MOI=2) for the indicated time and subjected to TEM to detect swollen phagosomes (open) using a previously validated method (Buter et al., 2019). Quantification was performed on cells from Day 1 (302 phagosomes), Day 3 (422 phagosomes), and Day 4 (232 phagosomes).

Supplemental Table 1 - Source of Cell lines, antibodies and chemicals used.

REAGENT or RESOURCE	SOURCE	IDENTIFIER
**Antibodies**		
CD206 – BV421	Biolegend	321125
CD16 – BV510	Biolegend	302048
HLA-DR – BV605	Biolegend	307639
CD36 – PE	Biolegend	336205
CD80 – PE-Cy7	BD Pharmingen	561135
CD14 – AF700	BD Pharmingen	557923
CD163 – PE-Texas Ted	Biolegend	333623
LC3a/b	Cell Signaling	4108S
LAMP1	Biolegend	328602
mTOR (7C10)	Cell Signaling	2983S
Goat anti-rabbit AF568	invitrogen	A11036
Goat anti- rabbit AF488	Life Technology	A10520
Goat anti- mouse AF568	invitrogen	A11031
Goat anti-mouse AF488	invitrogen	A11029
LC3B (immunofluorescence)	MBL	PM036
LAMP1 (murine) (immunofluorescence)	Developmental Studies	1D4B
LAMP1 (human) (immunofluorescence)	Developmental Studies	H4A3
LAMP1 (H5G11) (human) (immunofluorescence)	Santa Cruz Biotechnology	sc-18821
anti-Rabbit AlexaFluor 488 (immunofluorescence)	ThermoFisher	A21441
anti-rat AlexaFluor 568 (immunofluorescence)	ThermoFisher	A11077
anti-mouse AlexaFluor 568 (immunofluorescence)	ThermoFisher	A11004
anti-mouse AlexaFluor 594 (immunofluorescence)	ThermoFisher	A11005
LC3B (Western blot)	Abcam	ab48394
p62	CST	5114
Phospho-ULK1 (Ser757) (D7O6U)	CST	14202
ULK1 (D8H5)	CST	8054
Phospho-4E-BP1 (Ser65) (	CST	9452
4E-BP1	CST	9451
Phospho-p70 S6 Kinase (Thr389)	CST	9205
p70 S6 Kinase (49D7)	CST	2708
actin HRP	CST	12262
Mouse anti human LAMP1 (immunogold and CLEM)	Pharmingen	H4A3
Mouse anti human Perilipin2 (immunogold)	Progen	610102
Rabbit anti human LC3 (immunogold)	Abcam	
Anti Mtb cell wall protein (immunofluorescence)	John Spencer and Patrick Brennan	C188
Rabbit anti mouse bridging antibody (immonogold)	DAKO	Z259
Protein A 10 nm gold (immunogold)	Utrecht University	
Protein A 15nm gold (immunogold)	Utrecht University	
Goat anti mouse alexa 488 (CLEM)	Life technologies	A11001

Goat anti rabbit alexa 488	Molecular probes	A32731
**Human Cell Sources**<br><br>Leukoreduction Collars	 <br>BWH Blood Bank	
Leukocyte Cones	NHS Blood and Transport	
Bronchoalveolar lavage fluid	St James Hospital, Dublin	
**Chemicals, peptides, and recombinant proteins**		
Chloroquine diphosphate salt	Sigma Aldrich	C6628-100G
Oleate-BSA	Millipore Sigma	O3008-5ML
LPS	This lab	
hGMCSF	Peprotech	300-03-50UG
hMCSF	Peprotech	300-25-50UG
Glutaraldehyde	ThermoFisher/Electron Microscopy Sciences	50-262-18
Formaldehyde	ThermoFisher/Electron Microscopy Sciences	50-980-487
Saponin	Sigma Aldrich	
Paraformaldehyde	Sigma Aldrich	158127
Hoescht 33342 (bisBenzimide H 33342 trihydrochloride)	Sigma Aldrich	B2261
Fish Gelatin	Sigma Aldrich	G7765
**Critical commercial assays or reagents**		
DQ-BSA RED	Thermo Fisher/Invitrogen	D12051
C_{12}FDG	ThermoFisher Scientific - invitrogen	D2893
Bodipy 493/503	Thermo Fisher	D3922
LIVE/DEAD™ Fixable Blue Dead Cell Stain Kit	Thermo Fisher Scientific -invitrogen	L34962
MACS Milentyi LS columns	Miltenyi Biotec	130-042-401
$CD14^+$ beads	Miltenyi Biotec	130-050-201
Hoechst 33342	Life technologies	H21491
Rapamycin	Cell Signaling	9904S
LLOMe	Bachem	7000425
Monensin	Sigma	M5273
Bafilomycin A1	Sigma	B1793
HCS LipidTox Green	ThermoFisher Scientific - invitrogen	H34475
DAKO Fluorescent Mounting Medium	Agilent	S3023
**Cell lines**		
THP1	This lab/ATCC	
RAW264.7	Gutierrez lab/ATCC	
C8	Casma Therapeutics	
**Recombinant DNA**		
pJR966 CRISPRi plasmid – related to piJR965		Addgene #115163

GFP-mCherry-LC3B	Gift from Sharon Tooze, Francis Crick Institute, UK	
Software and algorithms		
Fiji		
GraphPad®Prism		
CellProfiler		
**Other**		
BD Difco™ Dehydrated Culture Media: Middlebrook 7H10 Agar	FisherScientific	BD 262710
Corning® 96 Well Solid Polystyrene Microplate	Millipore Sigma	CLS3917
RPMI 1640 Medium, no phenol red (Gibco)	Thermo FisherScientific	11835030
Zeocin™ Selection Reagent (Gibco)	Thermo FisherScientific	R25001
BD BBL™ Middlebrook OADC Enrichment	FisherScientific	B12351
BD Difco™ Dehydrated Culture Media: Middlebrook 7H9 Broth	FisherScientific	DF0713-17-9
BD Difco™ Glycerol	FisherScientific	DF0282-17-0
BD Difco™ Dehydrated Culture Media Additive: Tween™ 80 Polysorbate 80	FisherScientific	DF3118-15-6
RPMI 1640 Medium (Gibco)	ThermoFisher Scientific	52400025
Fetal Bovine Serum (Gibco)	ThermoFisher Scientific	10270106
Cefotaxime	Melford Biolaboratories Ltd.	
Amphotericin B (Gibco)	ThermoFisher Scientific	15290018
